# The Relationship Between Pediatric Gut Microbiota and SARS-CoV-2 Infection

**DOI:** 10.3389/fcimb.2022.908492

**Published:** 2022-07-08

**Authors:** Lorenza Romani, Federica Del Chierico, Gabriele Macari, Stefania Pane, Maria Vittoria Ristori, Valerio Guarrasi, Simone Gardini, Giuseppe Rubens Pascucci, Nicola Cotugno, Carlo Federico Perno, Paolo Rossi, Alberto Villani, Stefania Bernardi, Andrea Campana, Paolo Palma, Lorenza Putignani, the CACTUS Study Team, Francesca Calo` Carducci

**Affiliations:** ^1^ Infectious Disease Unit, Bambino Gesù Children’s Hospital, IRCCS, Rome, Italy; ^2^ Multimodal Laboratory Medicine Research Area, Unit of Human Microbiome, IRCCS, Bambino Gesù Children’s Hospital, IRCCS, Rome, Italy; ^3^ GenomeUp SRL, Rome, Italy; ^4^ Department of Diagnostic and Laboratory Medicine, Unit of Microbiology and Diagnostic Immunology, Unit of Microbiomics, Bambino Gesù Children’s Hospital, IRCCS, Rome, Italy; ^5^ Research Unit of Congenital and Perinatal Infections, Bambino Gesu` Children’s Hospital, IRCCS, Rome, Italy; ^6^ Chair of Pediatrics, Department of Systems Medicine, University of Rome ‘‘Tor Vergata’’, Rome, Italy; ^7^ Department of Diagnostic and Laboratory Medicine, Unit of Microbiology and Diagnostic Immunology, Multimodal Laboratory Medicine Research Area, Bambino Gesù Children’s Hospital, IRCCS, Rome, Italy; ^8^ Academic Department of Pediatrics, Bambino Gesu` Children’s Hospital, IRCCS, Rome, Italy; ^9^ Pediatric Emergency Department and General Pediatrics, Children Hospital Bambino Gesù, IRCCS, Rome, Italy; ^10^ Department of Pediatrics, Bambino Gesù Children’s Hospital, IRCCS, Rome, Italy; ^11^ Department of Diagnostic and Laboratory Medicine, Unit of Microbiology and Diagnostic Immunology, Unit of Microbiomics and Multimodal Laboratory Medicine Research Area, Unit of Human Microbiome, Bambino Gesù Children’s Hospital, IRCCS, Rome, Italy

**Keywords:** COVID-19, gut microbiota, dysbiosis, diversity index, SARS-CoV-2, immunology

## Abstract

This is the first study on gut microbiota (GM) in children affected by coronavirus disease 2019 (COVID-19). Stool samples from 88 patients with suspected severe acute respiratory syndrome coronavirus 2 (SARS-CoV-2) infection and 95 healthy subjects were collected (admission: 3–7 days, discharge) to study GM profile by 16S rRNA gene sequencing and relationship to disease severity. The study group was divided in COVID-19 (68), Non–COVID-19 (16), and MIS-C (multisystem inflammatory syndrome in children) (4). Correlations among GM ecology, predicted functions, multiple machine learning (ML) models, and inflammatory response were provided for COVID-19 and Non–COVID-19 cohorts. The GM of COVID-19 cohort resulted as dysbiotic, with the lowest α-diversity compared with Non–COVID-19 and CTRLs and by a specific β-diversity. Its profile appeared enriched in *Faecalibacterium*, *Fusobacterium*, and *Neisseria* and reduced in *Bifidobacterium*, *Blautia*, *Ruminococcus*, *Collinsella*, *Coprococcus*, *Eggerthella*, and *Akkermansia*, compared with CTRLs (*p <* 0.05). All GM paired-comparisons disclosed comparable results through all time points. The comparison between COVID-19 and Non–COVID-19 cohorts highlighted a reduction of *Abiotrophia* in the COVID-19 cohort (*p* < 0.05). The GM of MIS-C cohort was characterized by an increase of *Veillonella*, *Clostridium*, *Dialister*, *Ruminococcus*, and *Streptococcus* and a decrease of *Bifidobacterium*, *Blautia*, *Granulicatella*, and *Prevotella*, compared with CTRLs. Stratifying for disease severity, the GM associated to “moderate” COVID-19 was characterized by lower α-diversity compared with “mild” and “asymptomatic” and by a GM profile deprived in *Neisseria*, *Lachnospira*, *Streptococcus*, and *Prevotella* and enriched in *Dialister*, *Acidaminococcus*, *Oscillospora*, *Ruminococcus*, *Clostridium*, *Alistipes*, and *Bacteroides.* The ML models identified *Staphylococcus*, *Anaerostipes*, *Faecalibacterium*, *Dorea*, *Dialister*, *Streptococcus*, *Roseburia*, *Haemophilus*, *Granulicatella*, *Gemmiger*, *Lachnospira*, *Corynebacterium*, *Prevotella*, *Bilophila*, *Phascolarctobacterium*, *Oscillospira*, and *Veillonella* as microbial markers of COVID-19. The KEGG ortholog (KO)–based prediction of GM functional profile highlighted 28 and 39 KO-associated pathways to COVID-19 and CTRLs, respectively. Finally, *Bacteroides* and *Sutterella* correlated with proinflammatory cytokines regardless disease severity. Unlike adult GM profiles, *Faecalibacterium* was a specific marker of pediatric COVID-19 GM. The durable modification of patients’ GM profile suggested a prompt GM quenching response to SARS-CoV-2 infection since the first symptoms. *Faecalibacterium* and reduced fatty acid and amino acid degradation were proposed as specific COVID-19 disease traits, possibly associated to restrained severity of SARS-CoV-2–infected children. Altogether, this evidence provides a characterization of the pediatric COVID-19–related GM.

## Introduction

In late 2019, patients with coronavirus disease 2019 (COVID-19) were first recognized, and in early January 2020, the novel coronavirus severe acute respiratory syndrome coronavirus 2 (SARS-CoV-2) was identified as the etiologic agent of this new disease (Zhu et al., 2020). COVID-19 spread rapidly all over the world (Hui et al., 2020), and on March 12, 2020, the World Health Organization (WHO) declared a global pandemic (https://www.who.int/docs/default-source/coronaviruse/situation-reports/20200311-sitrep-51-covid-19).

SARS-CoV-2 through the angiotensin-converting enzyme 2 (ACE2) binds the epithelial cells in the respiratory tract, then starts replicating and migrating down to the airways, and enters to the alveolar epithelial cells in the lungs (Hu et al., 2021; Rezaei et al., 2021) with the risk of respiratory failure due to the strong immune response induced (Mehta et al., 2020; Ragab et al., 2020). Despite the respiratory symptoms are present in a major part of cases (Wu and McGoogan, 2020), gastrointestinal (GI) symptoms have been reported up to 20% in patients with COVID-19 (Cheung et al., 2020; Huang et al., 2020; Wu and McGoogan, 2020). Studies showed that ACE2 is expressed in ileum and colon, suggesting that the digestive tract organs also may be targeted by the virus (Xu et al., 2020; Zhang et al., 2020). SARS-CoV-2 RNA resulted as detectable in stool specimens from patients with COVID-19 (Chen et al., 2020a; Xie et al., 2020): up to 50% and 30% in adult and children, respectively, affected by the infection (De Ioris et al., 2020; Zuo et al., 2020). The virus was also found in the feces of patients with negative oral swab up to 23% of cases (Xiao et al., 2020), leading to the hypothesis that, in the intestine, there is not only a replication and activity but a greater permanence of the virus as well (Donati Zeppa et al., 2020).

SARS-CoV-2 infection reduces the expression of ACE2 in the GI tract and the number of circulating angiogenic cells (CACs), thus endangering the gut endothelium, leading to intestinal dysbiosis and then affecting the host immune response and metabolism (Chen et al., 2020a).

Therefore, the fecal microbiomes of patients with SARS-CoV-2 infection and its association with the infectivity and severity have been investigated.

Zuo et al. highlighted a profound alteration in fecal microbiomes of patient with COVID-19 characterized by enrichment of opportunistic pathogen and depletion of beneficial commensal. Patients with COVID-19 were depleted in gut bacteria with known immunomodulatory potential such as Faecalibacterium prausnitzii, Eubacterium rectale, and several bifidobacterial species; furthermore, the dysbiosis persisted even after clearance of SARS-CoV-2 (Xie et al., 2020; Zuo et al., 2020). More than one study reported an association between COVID-19 severity and intestinal microbiota (Xie et al., 2020; Zuo et al., 2020; Yeoh et al., 2021) and, recently, Rosario et al. proposed the gut microbiota (GM) diversity characterized by lower Firmicutes/Bacteroidetes ratio, higher abundance of Proteobacteria, and lower abundance of beneficial butyrate-producing bacteria such as the genera Roseburia and Lachnospira as prognostic biomarker of COVID-19 (Moreira-Rosário et al., 2021). According to that Reinold et al. found a GM of SARS-CoV-2–infected patients characterized by an enrichment of the phyla Proteobacteria and Bacteroidetes and a decrease of Actinobacteria compared with SARS-CoV-2–negative patients (Reinold et al., 2021).

The known modulation from the GM to the host immune response (Zheng et al., 2020) and the inflammatory condition described in COVID-19 (Yeoh et al., 2021) encouraged numerous studies to investigate the association between the GM and host inflammatory immune responses in COVID-19. Yeho et al. found that the composition of GM in patients with CODIV-19 was concordant with disease severity and magnitude of plasma concentrations of several inflammatory cytokines, chemokines, and blood markers tissue damage. All this evidence came from adult population; to date, there are no data about the GM profile in children affected by COVID-19.

A large variability in the susceptibility and in severity at different ages has been observed since the beginning of the pandemic (Lu et al., 2020a).

Children have been less severely involved than adults, requiring hospitalization only in 5%–10% of cases (Garazzino et al., 2020; Götzinger et al., 2020; Ludvigsson, 2020; Lu et al., 2020b). Most children infected by SARS-CoV-2 were asymptomatic or had mild symptoms, most commonly fever, cough, pharyngitis, GI symptoms, and changes in sense of smell or taste (Romani et al., 2020; Zachariah et al., 2020; Zimmermann and Curtis, 2021; Zimmermann and Curtis, 2022). Although the COVID-19 disease course was mild in children, a post-infectious inflammatory complication defined multisystem inflammatory syndrome in children (MIS-C) was described 2–4 weeks later from SARS-CoV-2 infection with a multiorgan involvement (Consiglio et al., 2020; Feldstein et al., 2021; McArdle et al., 2021; Dionne et al., 2022).

The potential role played by GM in COVID-19 in children has to be assigned, as it could be related to the mild course of the disease in children.

The aim of the present study was to examine the GM profile of pediatric patients with COVID-19 and to determine whether any imbalances of the commensal microbiota exist and, if so, whether they are correlated with disease severity.

## Materials and Methods

### Study Subjects and Design

Between March 1, 2020, and September 30, 2020, 88 children admitted in our hospital with signs and symptoms suspected of COVID-19 were consecutively enrolled in the study.

Patients with nasopharyngeal swab that tested positive for SARS-CoV-2 nucleic acid using reverse transcriptase qualitative polymerase chain reaction (PCR) assay were considered as confirmed cases of SARS-CoV-2 infection. Patients with negative swab tested for SARS-CoV-2 and diagnosis different from SARS-CoV-2 infection (16 patients) were included in the study and considered as a separate group (Non–COVID-19 cohort) as well as children affected by multisystem inflammatory syndrome (MIS-C). Patients with COVID-19 were stratified on the basis of disease severity according to the WHO clinical progression scale: 1) “mild”, if there was no evidence of viral pneumonia or hypoxia; 2) “moderate”, if there were clinical signs of non-severe pneumonia (cough or difficulty breathing, fast breathing, and/or chest indrawing) and no signs of severe pneumonia; 3) “severe”, if there was evidence of clinical signs of pneumonia (cough or difficulty in breathing) and at least one of the following: a) central cyanosis or SpO2 < 90%; severe respiratory distress; general danger sign: inability to breastfeed or drink, lethargy or unconsciousness, or convulsions; b) fast breathing (< 2 months: ≥ 60; 2–11 months: ≥ 50; 1–5 years: ≥ 40) (Agarwal et al., 2020).

Local ethical committee approved the study, and written informed consent was obtained from parents and legal guardians of all participants (2083_OPBG_2020). Age, gender, and clinical and routine laboratory characteristics were collected for each patient.

One-hundred thirty-two stool samples from all patients were collected at three time point: T_0_, close to the admission, within 48–72 h; T_1_, 72 h to 7 days from the admission; and T_2_, at the discharge; all stool samples were stored at −80°C.

All cohorts (COVID-19 and Non–COVID-19) were compared each other and with healthy controls (CTRLs). The CTRL cohort was composed by 95 healthy subjects, age-matched with patients, and selected by a survey provided by the OPBG Human Microbiome Unit on pediatric GM programming, in accordance with the recommendations of the OPBG Ethics Committee (Protocol No. 1113_OPBG_2016) approved on April 21, 2016.

### Detection of SARS-CoV-2 in Feces

Stool samples were resuspended in 1,000 µl of ASL Buffer, homogenized by vortexing, and processed by Allplex™ 2019-nCoV Assay on All-in-One Platform (Seegene, Korea). Supernatants (50 µl) were extracted using the STARMag Universal Cartridge kit (Seegene) on automated Nimbus IV platform and eluted in 100 µl of elution buffer. Real-time PCR was performed on CFX96 (Bio Rad Laboratories) with Allplex™ 2019-nCoV kit, using 5 µl of RNA in a final volume of 25 μl. An internal control was included in each sample for checking the extraction efficiency and PCR inhibition. In every experimental session, a negative control was used to monitor carryover contamination. The results were analyzed automatically using Seegene software (Seegene Viewer V2.0). Target genes were envelope (E) gene, RNA-dependent RNA polymerase gene/S gen (RdRP/S), and nucleocapsid (N) gene. Samples were considered positive when one or more genes were detected.

### Bacterial DNA Extraction From Stools and 16S rRNA Targeted Metagenomics

DNA from fecal samples of 132 stool samples from 88 patients with suspected SARS-CoV-2 infection and 95 CTRLs were extracted using the QIAmp Fast DNA Stool mini kit (Qiagen, Hilden, Germany), according to the manufacturer’s instructions. The 16S RNA targeted metagenomics was performed for all time point samples of COVID-19, Non–COVID-19, MIS-C, and CTRLs. Amplification of the variable regions V3–V4 from the bacterial 16S rRNA gene (∼460 bp) was carried out using the primers 16S_F 5′-(TCG TCG GCA GCG TCA GAT GTG TAT AAG AGA CAG CCT ACG GGN GGC WGC AG)-3′ and 16S_R 5′-(GTC TCG TGG GCT CGG AGA TGT GTA TAA GAG ACA GGA CTA CHV GGG TAT CTA ATC C)-3′ ++ (Illumina, San Diego, California, USA). The PCR reaction was set up using the 2× KAPA Hifi HotStart ready Mix kit (KAPA Biosystems Inc., Wilmington, Massachusetts, USA). DNA amplicons were cleaned up by CleanNGS kit beads (CleanNA, Coenecoop 75, PH Waddinxveen, The Netherlands). A second amplification step was performed to obtain a unique combination of Illumina Nextera XT dual-indices for each sample. The final libraries were cleaned up using CleanNGS kit beads, quantified by the Quant-iT PicoGreen dsDNA Assay Kit (Thermo Fisher Scientific, Waltham, Massachusetts, USA), and normalized to 4 nM. The following steps consisted of samples’ pooling, denaturation, and dilution to 7pM. To generate paired-end 250 × 2-bp length reads, normalized libraries were pooled together and run on the Illumina MiSeqTM platform, according to the manufacturer’s specifications.

### Biocomputational and Statistical Analysis for GM Profile Analysis and Patients’ Metadata Correlation

Paired-end sequencing reads in fastq format were analyzed using QIIME2 (Bolyen et al., 2019). Hence, samples characterized by reads number <1,000 and >150,000 were excluded, resulting in 200 samples. The QIIME2 plugin for DADA2 (Callahan et al., 2016) was used for quality control, denoising, chimera removal, trimming, and construction of the Amplicon Sequence Variant (ASV) table. The taxonomy was assigned by using a Naive Bayes model pre-trained on Greengenes 13_8 (Khan et al., 2021; Kang et al., 2022) through the QIIME2 plugin q2-feature classifiers (Bokulich et al., 2018). Alpha- and beta-diversity were computed by skbio.diversity using analysis of variance (ANOVA test) and permutational analysis of variance (PERMANOVA test), respectively; the latter was applied on phylogenetically informed weighted and unweighted Unifrac, Bray–Curtis, and Euclidean distance matrices (Lozupone and Knight, 2005; Chen et al., 2012) with 9,999 permutations to perform paired-comparison of COVID-19, Non–COVID-19, and CTRL samples at different time points. Principal coordinate analysis plots were used to illustrate the beta-diversity of samples.

The ASV table was normalized using the Cum Sum Scaling (CSS) methodology (Paulson et al., 2013); hence, the Kruskal–Wallis test was applied to compare taxonomic differences at the phylum (L2), family (L5), and genus (L6) levels. Unless otherwise stated, all ecological statistical analyses were performed using Python 3.7.

Three different levels of statistical significance were identified based on different *p*-values (*p* ≤ 0.001) and false discovery rate (FDR) thresholds (*p* ≤ 0.05, *p* ≤ 0.001) (Benjamini and Hochberg, 1995).

Phylogenetic Investigation of Communities by Reconstruction of Unobserved States (PICRUSt), exploiting the Kyoto Encyclopedia of Genes and Genomes (KEGG) orthologs (KO) database was used to determine ASVs and their microbiome’s functional potential to characterize microbial populations of COVID-19 and age-matching CTRL groups.

### Inflammatory and Immunological Blood Proteins

To study the relationship between inflammatory and immunological blood proteins and ASVs in the GM of patients with COVID-19, we performed an unsupervised clustering analysis at L2, L5, and L6, selecting disease severity as feature for correlation.

With this aim, 184 blood biomarkers of immune response and inflammation for the 68 patients with COVID-19 were assayed by a multiplex technology based on proximity extension assays (Chun and KeleÅ, 2010), using two Olink panels, each containing 96 pairs of DNA-labeled antibody probes (Olink AB, Uppsala, Sweden).

To minimize inter- and intra-run variation, the data were normalized using both an internal control (extension control) and an inter-plate control and then transformed using a pre-determined correction factor. The pre-processed data were provided in the arbitrary unit Normalized Protein Expression (NPX) on a log2 scale and where a high NPX represents high protein concentration.

Statistical analysis related to correlation between ASV abundances and protein levels was performed using R software (version 3.6.2). ASVs with a number of zeros greater than 70% were omitted from the analysis. PCAs were computed using the prcomp R function on log2-transformed abundances values. For the unsupervised clustering analysis, ASV abundances were transformed to log2 scale. In correlation heatmaps, Spearman’s correlation was used to examine the association between features and only statistically significant correlations (FDR adjusted p-values < 0.05). The R package “enrichR” v3.0 was used to perform pathway enrichment analysis in Reactome 2016, KEGG 2021 (Human), GO Biological Process 2021, and GO Molecular Function 2021 databases on proteins positively or negatively associated to ASV abundance. Positive association between ASV and proteins was selected according to mean Spearman correlation index (rho) >0 or <0 in negatively associated proteins.

### Clustering Analysis and Machine Learning

In heatmaps based on hierarchical clustering of COVID-19, CTRL subgroups, and ASVs, KOs were performed by Pearson correlation distance. Data dimensionality reduction, based on Principal component analysis (PCA) and partial least-squares regression (PLS), was used to represent COVID-19 and CTRL interpretable visualization of KO feature projection. As an initial analysis, some statistics were performed on ASV tables (L2–L6). The interquartile range (IQR), the percentage of zeros (%0), the fold change (FC), and the T-test with relative p-value (corrected with the FDR method) were calculated. Applying these filters: IQR ≠ 0, %0 ≥ 75%, and FC ≠ 0, the variables that were totally unnecessary for classification were discarded. After applying the filters, further statistics were performed on the remaining variables: for each ASV, the count of patients in each class with abundances > 0, mean and standard deviation of the abundances, and a Z-test with relative p-value between the two classes. To have a global view of the data, at each level, a Z-score score heatmap along the abundances of the organisms was performed. Dimensionality reduction of data was represented by projection methodologies PCA and sparse partial least-squares regression (SPLS) (Chun and KeleÅ, 2010).

Multiple machine learning (ML) models were trained for the classification tasks. The pipeline consists of a 10-fold cross-validation with a train-test split of 70%–30%. To evaluate the model, the global and the single-class accuracies were considered. The models tested were Logistic Regression, SGD Classifier, Logistic Regression CV, Hist Gradient Boosting Classifier, Random Forest Classifier, Extra Trees Classifier, Gradient Boosting Classifier, Bagging Classifier, Ada Boost Classifier, XGB Classifier, XGBRF Classifier, MLP Classifier, Linear SVC, SVC, Gaussian NB, Decision Tree Classifier, Quadratic Discriminant Analysis, K Neighbors Classifier, and Gaussian Process Classifier. An explain ability algorithm based on a permutation performance with 1,000 repetitions was followed.

A Spearman correlation analysis between ASV and KOs was performed for COVID-19 and CTRLs, selecting the features with importance value >0 from the feature importance analysis.

### Metagenomic Data Open Access Repository

All raw sequencing reads are available at NCBI BioProject database (PRJNA753792) (https://submit.ncbi.nlm.nih.gov/subs/sra/) (accessed on September 30, 2022).

## Results

### Patient Cohort and Stratification

Sixty-eight children with confirmed SARS-CoV-2 infection (i.e., COVID-19) and 20 patients with negative SARS-CoV-2, including 16 subjects affected by other diagnosis than SARS-Cov-2 (i.e., Non–COVID-19) and four subjects affected by multisystem inflammatory syndrome (MIS-C), were included in this observational cohort study.

Demographic and clinical characteristics of COVID-19, Non–COVID-19, and MIS-C cohorts are reported in **Table 1**.

**Table 1:** Demographic and clinical features at the admission.

Variables	COVID-19	Non–COVID-19	MIS-C
**Number of patients**	68	16	4
**Age**			
Median years (IQR)	6.5 (0.9–11.5)	4.4 years (1.7–6.3)	11.7 years (10.5–12.8)
Min	8 days	1 years	8 years
Max	17 years	16 years	14.5
**Sex**			
Male	38 (56%)	10 (53%)	3 (75%)
**Comorbidities**	4 (6%)	5 (26%)	1 (25%)
**Symptoms at the admission**			
Fever	36 (56%)	15 (79%)	4 (100%)
**Respiratory symptoms**			
Cough	18 (27%)	5 (26.3%)	0
Shortness of breath	7 (10%)	0	0
Rhinorrhea	2 (3%)	1 (5.2%)	0
**Gastrointestinal symptoms**			
Diarrhea	11 (16%)	4	1 (25%)
Vomit	3 (4%)	1	1 (25%)
Disease severity			
Asymptomatic	12 (18%)	NA	NA
Mild	49 (72%)	NA	NA
Moderate	7 (10%)	NA	NA
**Blood result**			
Lymphopenia	11 (22%)	1(5.2%)	2 (50%)
C reactive protein, median, mg/dl	0.09	4.81	6
**Radiological results**			
Suggestive of viral pneumonia	7 (10%)	NA	NA
**Coinfection**			
Viral coinfection	1 (HHV6)	NA	NA
Bacterial coinfection	4 (6%)	NA	NA
Antibiotic use	18 (26%)	10 (63%)	4 (100%)

There were 38 male and 30 female patients with COVID-19 with a median age of 6.5 years. Among them, five were affected by a coinfection: one had Human Herpes Virus 6 (HHV6), two had a urinary tract infection (UTI), one had gastroenteritis by *Campylobacter jejuni*, and another one had an otitis media.

In the COVID-19 cohort, four children had comorbidities such as genetic syndrome, autism, Kikuchi syndrome, and connective tissue disorder.

Only 18 (26%) patients affected by SARS-CoV-2 infection received antibiotic therapy during the admission.

The Non–COVID-19 cohort included 10 male (62%) and six female (38%) patients with a median age of 4.4 year. In this cohort, nine patients had lower and upper respiratory infections (LRI and URI), two had GI infection, three received diagnoses of HHV6, and two were affected by non-infectious disease (epilepsy and hypoglycemia) (**Table 1**).

The MIS-C cohort included four patients, three were male (75%), and the median age was 11.7 years. No one of them had comorbidities and no one had a coinfection (**Table 1**).

### GM Profiling of Children With COVID-19

In total, 106 stool samples were collected from patients with SARS-CoV-2 infection (at T_0_ = 68, at T_1_ = 27; at T_2_ = 11). The GM composition of patients with COVID-19 was compared with Non–COVID-19 and CTRL cohorts, whereas MIS-C cohort was not included in the ecological analyses for the scarceness of samples. The total number of collected specimens is reported in **Table S1**. The number of reads for each sample is reported in **Table S2**.

### Correlation Between Clinical Features and GM Global Distribution

**Figure 1:**
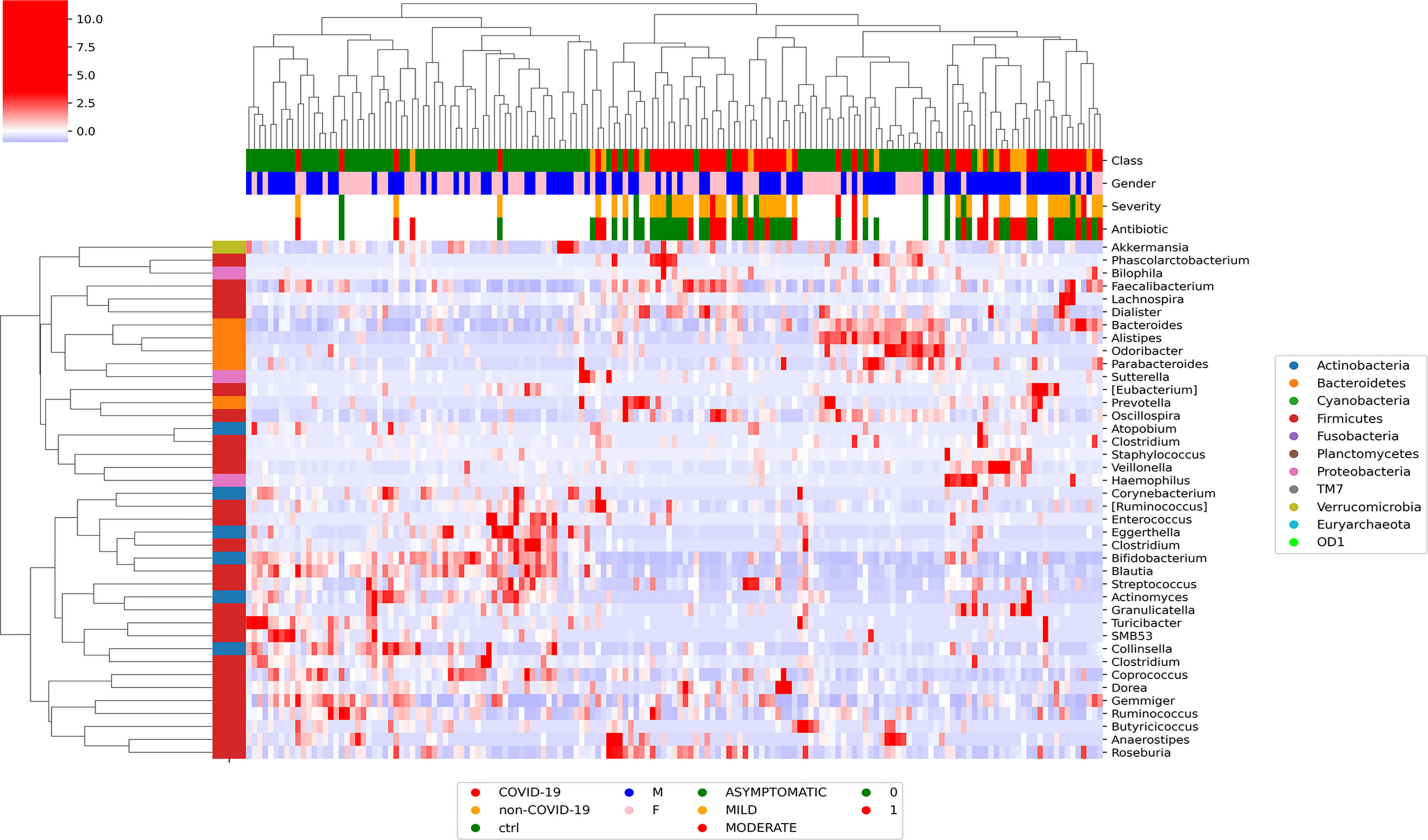
Graphical representation of hierarchical analysis of global ASV distribution at L6 (genus) for COVID-19 and CTRL subjects filtered by a t-test between classes with *p*-value < 0.05 at T_0_. In the heatmap, the hierarchical complete linkage dendrogram is based on the ASVs Pearson’s correlation coefficient. The color scale characterizes the Z-score for each variable: red, high level; blue, low level. The column color labels represent respectively: patient’s class (red, COVID-19; orange, Non–COVID-19; green, CTRLs), gender (blue, male; pink, female), severity (green, asymptomatic; orange, mild; red, moderate), and antibiotic [green, absent (0); red, present (1)].

At L2, Pearson’s correlation revealed that COVID-19 samples showed a significant correlation with more group of ASVs. In particular, Proteobacteria were positively correlated with patients affected by SARS-CoV-2 infection, whereas Actinobacteria were negatively correlated with the infection (**Figure S1**). At L5, the hierarchical cluster showed that Veillonellaceae, Staphylococacceae, Desulfovibrionaceae, Ruminococcaceae, and Prevotellaceae positively correlated with COVID-19, whereas Enterococcaceae, Bifidobacteriaceae, Coriobacteriaceae, Actinomycetaceae, and Lachnospiraceae were negatively correlated (**Figure S2**). At L6, *Faecalibacterium*, *Phascolarctobacterium*, *Dialister*, *Roseburia*, *Ruminococcus*, and *Prevotella* were positively correlated with SARS-CoV-2 infection, whereas *Blautia*, *Actinomyces*, *Bifidobacterium*, *Collinsella*, *Turicibacter*, *Sutterella*, and *Alistipes* were negatively correlated with the disease (**Figure 1**).

### Gut Microbiota Diversity Analyses of COVID-19 and Non–COVID-19 Cohorts and Healthy Subjects

**Figure 2:**
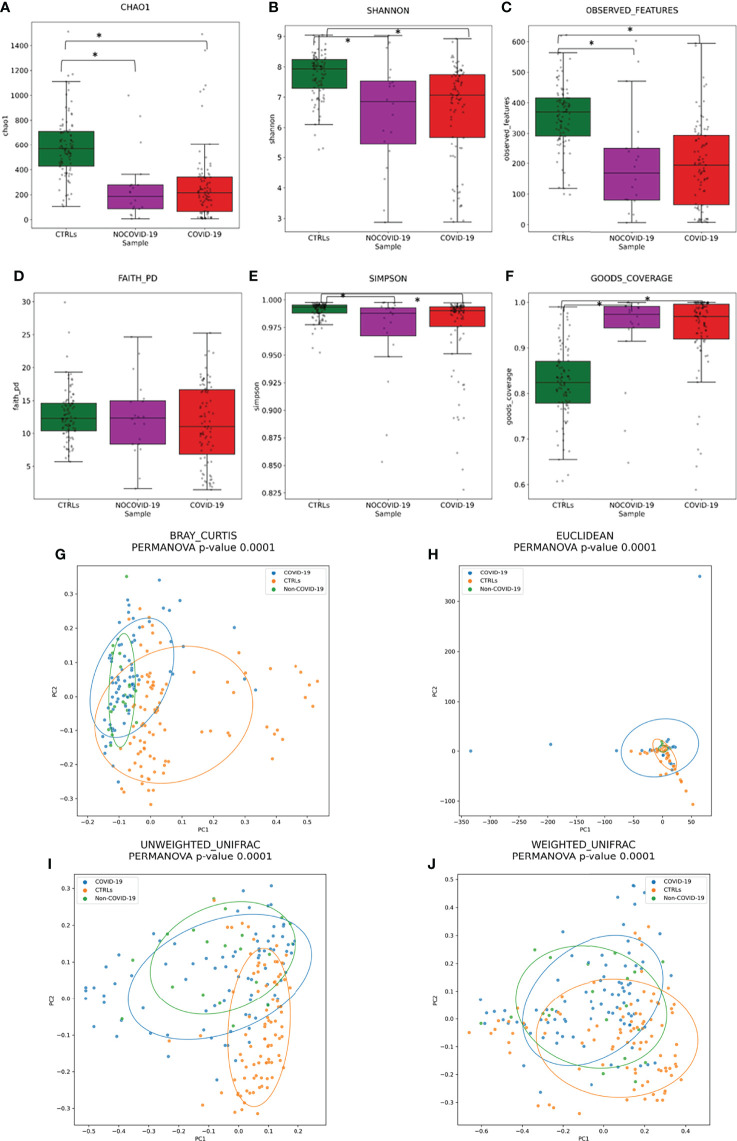
Gut microbiota ecology evaluation. Alpha-diversity of COVID-19 and Non–COVID-19 cohorts and CTRLs based on Chao-1 **(A)**, Shannon **(B)**, observed species **(C)**, Faith PD **(D)**, Simpson **(E)**, and Good’s coverage **(F)** indices. Statistically significant (*p*-value < 0.05) comparisons by ANOVA test are indicated by asterisk. Beta-diversity of COVID-19 and Non–COVID-19 cohorts and CTRLs, performed by Bray–Curtis **(G)**, Euclidean distance **(H)**, and unweighted **(I)** and weighted **(J)** UniFrac algorithms.

To assess the overall differences of microbial community structures, we evaluated the diversity among COVID-19 and Non–COVID-19 cohorts and healthy CTRLs using the α-diversity including Chao-1, Shannon, observed species, phylogenetic distance, Good’s coverage, and Simpson indexes (**Figure 2**).

A significant reduction in α-diversity was observed in COVID-19 cohort compared with healthy subject (*p*-value < 0.05), using Chao-1, Shannon, observed species, and PD phylogenetic distance indexes (**Figures 2A–F**), as well as between Non–COVID-19 cohort and healthy subject (*p*-value < 0.05). There was no significant difference between COVID-19 cohort and Non–COVID-19 cohort (*p*-value > 0.05).

Beta-diversity analyses, performed by Bray–Curtis, Euclidean distance, and unweighted and weighted UniFrac algorithms, showed the COVID-19 and Non–COVID-19 cohorts separated in clusters from CTRLs (*p-*value < 0.05 for all the analyses, PERMANOVA) (**Figures 2H–J**).

### Gut Microbiota Global Composition of COVID-19 and Non–COVID-19 Cohorts and Healthy Subjects

**Figure 3:**
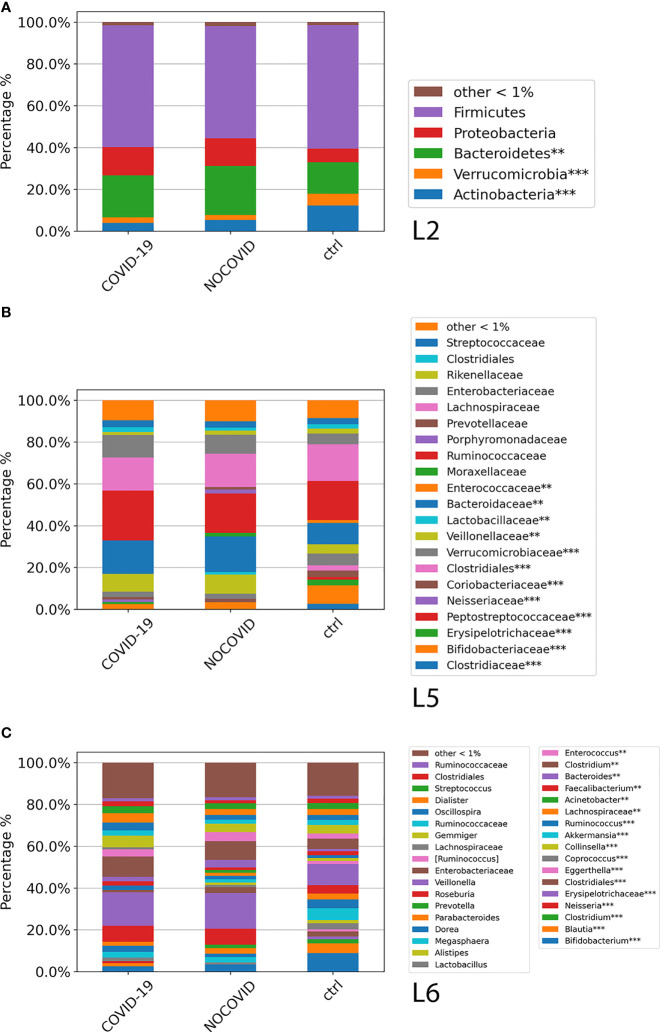
ASV distributions of COVID-19 and Non–COVID-19 cohorts and CTRLs at L2 **(A)**, L5 **(B)**, and L6 **(C)**, filtered by statistically significance based on the Kruskal–Wallis test. *p-value < 0.001; *p*-value **FDR < 0.05; ***p-value FDR < 0.001.

The GM global composition of the study cohorts at L2, L5, and L6 levels is shown in **Figures 3A–C** and **Table S3**. From the relative distribution at phylum level, all the study cohorts were dominated by five phyla: Firmicutes, Bacteroidetes, Proteobacteria, Actinobacteria, and Verrucomicrobia. The Firmicutes was the predominant phylum contributing about to the 55%–60% of the GM in each cohort followed by Bacteroidetes, which contributed to the Non–COVID-19 cohort, COVID-19 cohort, and CTRL GM of 23%, 20%, and 15%, respectively. Proteobacteria resulted as the third dominant phylum in the COVID-19 GM (14%) and Non–COVID-19 GM (13%), whereas in the CTRL GM, this phylum represented only 6%. Actinobacteria was the fourth most represented phylum in the GM of COVID-19 (4%) and Non–COVID-19 (5%), whereas was significantly increase (FDR < 0.05) in the CTRL GM (12%). Verrucomicrobia was more represented (FDR < 0.05) in CTRLs (5.6%) than COVID-19 (2.7%) and Non–COVID-19 cohorts (2.5%). The composition of the intestinal microbiota at L2 is reported in **Figure 3A**.

The analysis of ASV distribution at L5 showed a higher abundance of Ruminococcaceae (24%) in the GM of COVID-19 compared with the GM of Non–COVID-19 cohort (18.8%) and CTRLs (18.7%). Bacteroidaceae, Neisseriaceae, Verrucomicrobiaceae, and Veillonellaceae were significantly increased (FDR < 0.05) in the GM of COVID-19 and Non–COVID-19 cohorts compared with the CTRLs (**Figure 3B**). A significant reduction (FDR < 0.05) of Bifidobacteriaceae in COVID-19 and Non–COVID-19 cohorts compared with CTRLs (2.5%, 3.4%, and 8.8%) was observed (**Figure 3B**).

At L6, a significant reduction of *Bifidobacterium*, *Blautia*, and *Akkermansia* in the GM of COVID-19 and Non–COVID-19 cohorts compared with CTRLs was observed, whereas a significant increase (FDR < 0.05) of *Faecalibacterium*, *Bacteroidetes*, and *Neisseria* was characterized in the GM of COVID-19 and Non–COVID-19 cohorts compared with the CTRLs (**Figure 3C**).

An insight onto GM coupled comparisons, also filtered by the Kruskal–Wallis test, was reported for COVID-19 versus CTRLs, and COVID-19 versus Non–COVID-19, including stool samples collected at T_0_ and at the following time points.

### Gut Microbiota Global Composition of COVID-19 Cohort Compared With Healthy Subjects

**Figure 4:**
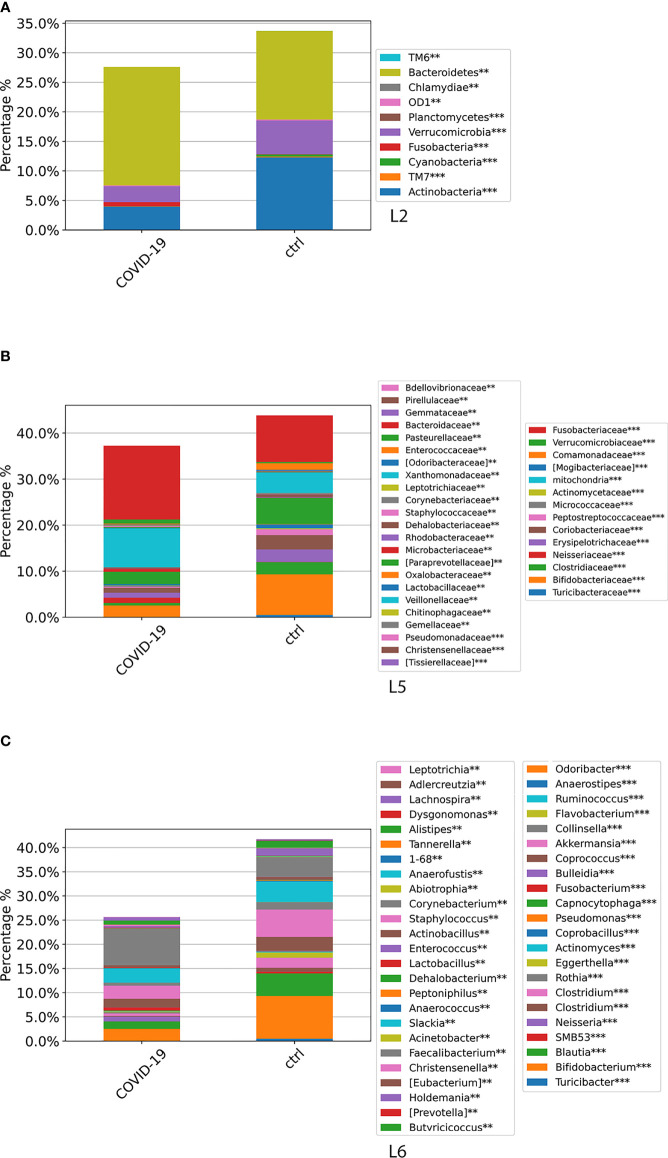
ASV distributions at L2 **(A)**, L5 **(B)**, and L6 **(C)**, filtered by statistically significance based on the Kruskal–Wallis test. *p-value < 0.001; *p*-value **FDR < 0.05; ***p-value FDR < 0.001. ASV distributions were reported in the comparisons among COVID-19 and CTRL subjects.

At L2, a significant difference (*p*-value FDR < 0.001) for both Actinobacteria and Verrucomicrobia was observed, resulting in decreased in the GM of COVID-19 cohort compared with CTRLs, whereas Fusobacteria and Bacteroidetes were significantly increased in the GM of COVID-19 (*p*-value FDR < 0.001 and *p*-value FDR < 0.05, respectively) (**Figure 4A**). At L5, significant differences were found between GM of COVID-19 cohort and CTRLs: an increase of Veillonellaceae (*p*-value FDR < 0.001), Neissereriaceae, Bacteroidaceae, and Fusobacteriaceae (*p*-value FDR < 0.05) was observed in the GM of COVID-19 cohort, whereas a significant reduction was found for Bifidobacteriaceae (*p*-value FDR < 0.001), Coriobacteriaceae (*p*-value FDR < 0.001), Peptostreptocococcaceae (*p*-value FDR < 0.001), Clostridiaceae (*p*-value FDR < 0.001), and Verrucomicrobiaceae (*p*-value FDR < 0.001) (**Figure 4B**). At L6, the GM of COVID-19 cohort was significantly different from the GM of CTRLs, with an increase of *Faecalibacterium* (*p*-value FDR < 0.05), *Fusobacterium* (*p*-value FDR < 0.001), and *Neisseria* (*p*-value FDR < 0.001) and a reduction of *Bifidobacterium* (*p*-value FDR < 0.001), *Collinsella* (*p*-value FDR < 0.001), *Eggerthella* (*p*-value FDR < 0.001), *Blautia* (*p*-value FDR < 0.001), *Coprococcus* (*p*-value FDR < 0.001), *Ruminococcus* (FDR < 0.001), and *Akkermansia* (*p*-value FDR < 0.001) (**Figure 4C**).

### Gut Microbiota of Patients With COVID-19 at the Admission Time

To investigate whether SARS-CoV-2 might affect GM composition since the first days of the infection, α- and β-diversity and ASV distribution were determined for the COVID-19 only including stool samples collected at T_0_, identified as the time point within 48–72 h since admission.

Alpha-diversity appeared statistically reduced for COVID-19 compared with CTRLs (**Figure S3**). Beta-diversity, computed by Bray–Curtis, Euclidean distance, and unweighted and weighted UniFrac algorithms, showed a clear separation of COVID-19 and CTRLs (*p*-value < 0.0001 for all the analyses, PERMANOVA) (**Figure S4**).

**Figure 5:**
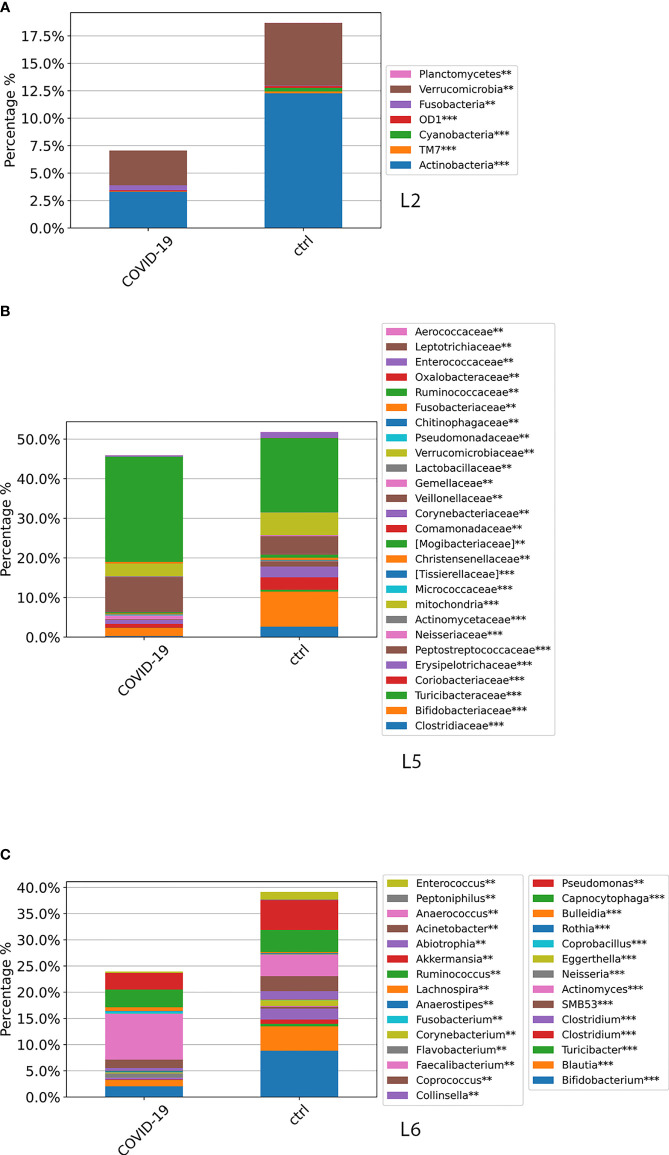
ASV distributions at L2 **(A)**, L5 **(B)**, and L6 **(C)** filtered by statistically significance based on the Kruskal–Wallis test. *p-value < 0.001; *p*-value **FDR < 0.05; ***p-value FDR < 0.001. ASV distributions were reported in the comparisons among COVID-19 and CTRL subjects at the admission time.

Global ASV distribution of COVID-19 cohort, compared with CTRLs, at L2, L5, and L6 levels is shown in **Figure 5**. The GM profiling comparison disclosed the same results obtained through all time points, as reported in **Figure 4**.

Furthermore, we compared the GM composition of stool samples collected at T_0_ vs. T_1_ and T_2_. As shown in **Figure S7**, we observed similar results between GM profile at the admission (T_0_) and during the hospitalization (T_1_ and T_2_) except for slight differences for species with low abundance (**Figure S5**).

### Gut Microbiota Global Composition of COVID-19 Compared With Non–COVID-19 Cohorts

In the comparison between COVID-19 and Non–COVID cohorts at L2, Firmicutes appeared increased, whereas Proteobacteria and Bacteroidetes decreased; at L5, a higher abundance of Neisseriaceae, Veillonellaceae, Ruminococcaceae, and Streptococcaceae and a lower abundance of Lactobacillaceae and Coriobacteriaceae were observed in the COVID-19 cohort compared with Non–COVID-19 cohort. At L6, the ASV distribution showed a slight prevalence of *Klebsiella*, *Neisseria*, *Dorea*, *Roseburia*, *Oscillospora*, and *Dialister* in the COVID-19 cohort and a reduction of *Abiotrophia* (statistically significant only evidenced in the comparison between COVID-19 and Non–COVID-19 cohorts at T_0,_ p < 0.05), *Parabacteroides*, and *Clostridium* (data not shown).

### Gut Microbiota Global Composition of MIS-C Compared With Healthy Subjects

The MIS-C cohort GM was characterized by an increase of Fusobacteria compared with CTRLs at L2 (**Figure S6A**); at L5, Neisseriaceae, Fusobacteriaceae, Veillonellaceae, and Ruminococcaceae were more abundant in the MIS-C (**Figure S6B**). Looking at L6 the analysis showed a MIS-C GM rich in *Veillonella*, *Clostridium*, *Dialister*, *Ruminococcus*, and *Streptococcus* and poor of *Bifidobacterium*, *Blautia*, *Granulicatella*, and *Prevotella* (**Figure S6C**).

### Gut Microbiota of Patients With COVID-19 With a Coinfection

The COVID-19 cohort included five patients (11 stool samples) affected by a coinfection (COVID-19 ^++^); therefore, we investigated their GM compared with that of patients with COVID-19 without coinfections. Alpha-diversity for COVID-19 ^++^ showed the same trend of COVID-19 (**Figure S7**). Beta-diversity showed COVID-19, COVID-19 ^++^, and Non-COVID cohorts and CTRLs well separated in clusters (PERMANOVA, p = 0.001) (data not shown). Looking at the ASV distribution for COVID-19^++^ and COVID-19 cohorts, a significant difference (*p*-value FDR < 0.05) was observed at L2, with Proteobacteria less abundant in the COVID-19^++^ compared with the COVID-19; at L5, Enterobacteriaceae appeared reduced in the COVID-19^++^ (*p*-value FDR < 0.05) (data not shown). No significant differences were found at L6.

### Gut Microbiota Profile Associated to COVID-19 Severity

As severity measure the WHO scale was exploited (Agarwal et al., 2020). In our COVID-19 cohort, 12 patients (16%) were asymptomatic, 49 children (73%) were classified as “mild”, seven patients (11%) had a “moderate” disease, and no case was defined as “severe”.

Pearson’s correlation revealed that COVID-19 severity did not present any correlation at L2, L5, and L6 with one or more specific ASVs (**Figures S1**, **S2** **;** **1**).

An overall reduced α-diversity was observed in “mild” and “moderate” compared with the “asymptomatic” group. In detail, a reduction of α-diversity was observed by comparing the “mild” with the “asymptomatic” group based on Chao-1, observed species, and Good’s coverage indexes (p-< 0.05) **(** **Figure S8** **)**.

Despite not statistically significant, three clusters (“asymptomatic”, “mild”, and “moderate”) were identified by β-diversity (data not shown).

The ASV distribution did not highlight any statistically significant difference at L2, L5, and L6. However, looking at ASV distribution (**Figure S9**), the GM of the “moderate” group was characterized at L2 by a reduction of Proteobacteria and Actinobacteria and by an increase of Bacteroidetes; at L5 by a reduction of Bifidobacteriaceae, Streptococcaceae, and Enterobacteriaceae and an increase of Bacteroidaceae (**Figure S9**); and at L6 by an higher abundance of *Dialister*, *Acidaminococcus*, *Oscillospira*, *Ruminococcus*, *Clostridium*, *Alistipes*, and *Bacteroides* and a lower distribution of *Neisseria*, *Lachnospira*, *Streptococcus*, and *Prevotella* **(** **Figure S9** **)**.

### Effect of Antibiotics on GM of COVID-19 Cohort

The percentage of patients who received antibiotic was higher in the “moderate” group (71%) compared to the “mild” (0%) and asymptomatic (9%) groups. To investigate whether the administration of antibiotics could drive a modulation of the GM and therefore affect the ecology, the COVID-19 cohort was categorized on the basis of antibiotic administration during hospital stay (yes = 1 and no = 0). No statistically significant differences were reported for α-diversity, whereas, for the β-diversity computed by Bray–Curtis algorithm, cluster separation was clear (p < 0.05, PERMANOVA), regardless of no significant separation was assessed using Euclidean and unweighted and weighted UniFrac algorithms (p > 0.05, PERMANOVA) **(** **Figure S10** **)**. The ASV distribution highlighted a significant reduction of Veillonellaceae at L5 in the subgroup of patients with COVID-19 receiving antibiotics (**Figure S11**).

### Effect of SARS-Cov-2 Positivity in Feces on GM of COVID-19 Cohort

Among the 106 stool samples analyzed for the COVID-19 cohort, 55 tested positive for SARS-CoV-2 (52%). Therefore, we investigated whether the presence of the virus in feces was possibly associated with changes in the GM profile. As depicted in **Figure S12**, no statistically significant differences were detected for α-diversity indices for the SARS-CoV-2–positive group. Beta-diversisty analyses highlighted a group separation by Euclidean distance matrix. In addition, the ASV distribution did not highlight any statistically significant difference at L2, L5, and L6. However, looking at ASV distribution at L6 in the “positive SARS-CoV-2”–related GM, a slight increase of *Prevotella*, *Streptococcus*, *Lachnospira*, and *Veillonella* and a reduction of *Klebsiella*, *Ruminococcus*, *Clostridium*, and *Lactobacillus* were observed compared with the “negative SARS-CoV-2” stool samples (**Figure S13**).

### Model Classifications Analysis

**Figure 6:**
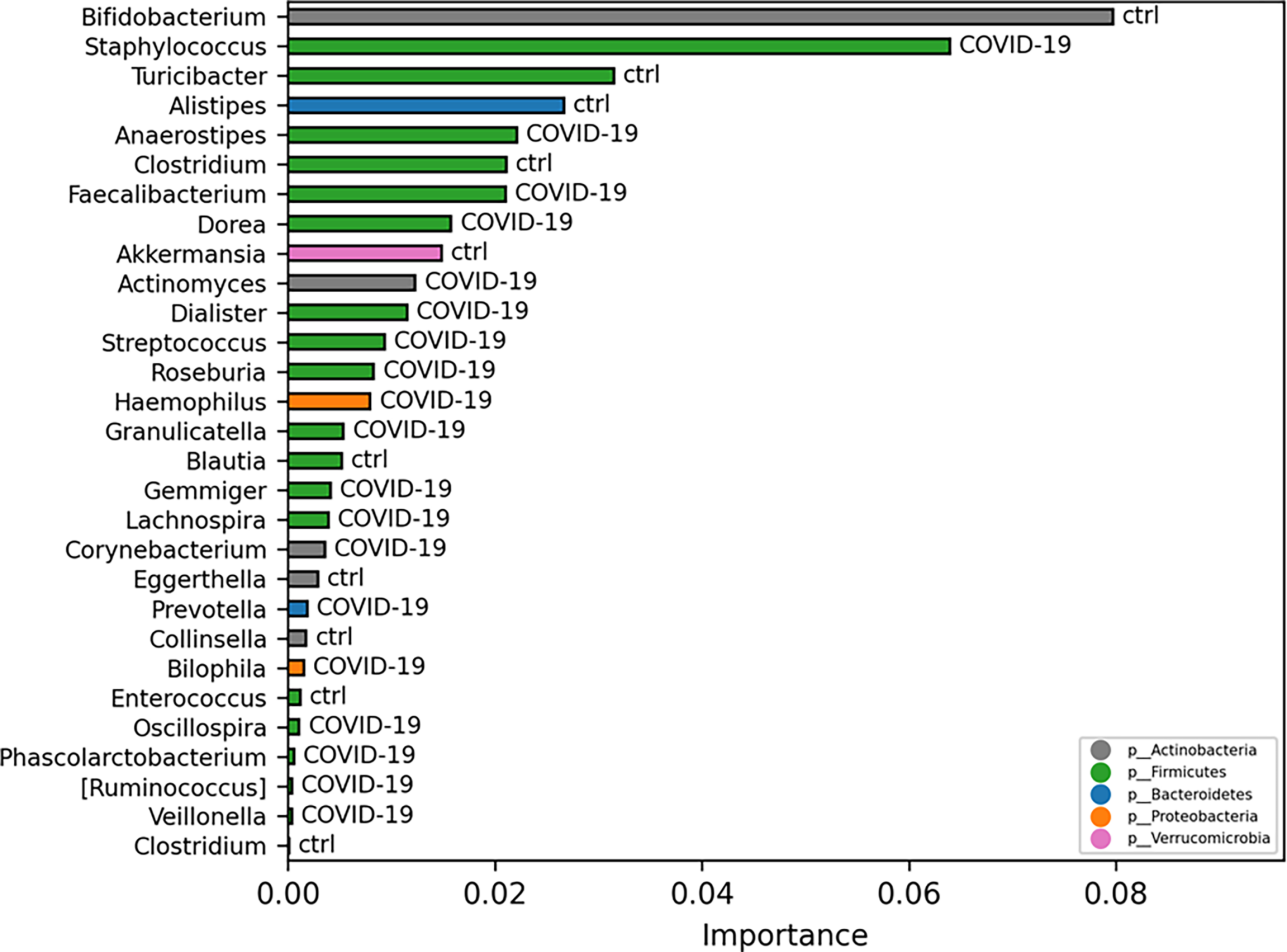
Important ASVs selected by model classification analysis. The bars represent the importance scores of each ASV in the prediction of models.

Exploiting ML model for GM biomarker prediction in patients with COVID-19, compared with CTRLs, the disease-associated microbial markers at L6 are as follows: *Staphylococcus*, *Anaerostipes*, *Faecalibacterium*, *Dorea*, *Dialister*, *Streptococcus*, *Roseburia*, *Haemophilus*, *Granulicatella*, *Gemmiger*, *Lachnospira*, *Corynebacterium*, *Prevotella*, *Bilophila*, *Phascolarctobacterium*, *Oscillospira*, and *Veillonella*, representing in the model prediction the range 0.02–0.004 of importance score (**Figure 6** **)**.

Furthermore, to investigate whether GM profile could be predictive of COVID-19 disease, the ML algorithm was exploited. This analysis, at L6, revealed that the microbiota had capability to classify the 90% of the COVID-19 and CTRLs by the models LogisticRegression, LogisticRegressionCV, HistGradientBoostingClassifier, RandomForestClassifier, GradientBoostingClassifier, AdaBoostClassifier, MLPClassifier, LinearSVC, and DecisionTreeClassifier (**Table S4**).

### COVID-19 GM-Predicted Function

**Figure 7:**
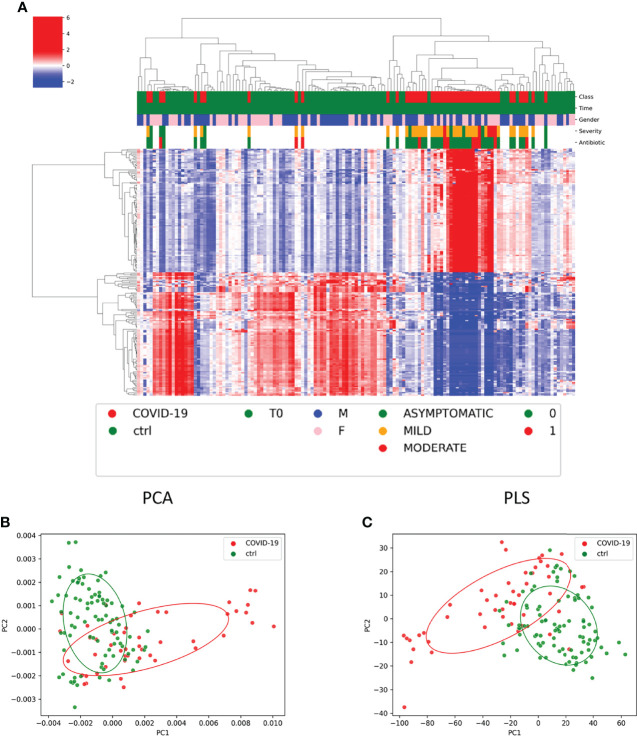
Functional profile of microbial communities of COVID-19 and CTRL subgroups. **(A)** Graphical representation of hierarchical analysis of the KO-based prediction of functional profile of microbial communities of COVID-19 and CTRL subgroups. The top 200 features with lowest *p*-value (p < 0.05). The color scale characterizes the Z- score for each variable: red, high level; blue, low level. The column color labels represent respectively: patient’s class (red, COVID-19; green, CTRLs), time (green, T_0_), gender (blue, male; pink, female), severity (green, asymptomatic; orange, mild; red, moderate), and antibiotic [green, absent (0); red, present (1)]. **(B, C)** Principal component analysis (PCA) and partial least-squares regression (PLS) interpretable visualizations, based on of KO feature projection for COVID-19 and CTRLs.

The KO-based prediction of functional profile of microbial communities of COVID-19 and CTRL subgroups highlighted 2,148 KO classification features (**Figure S14**). To have more detailed view, the top 200 features with lowest *p*-value (p < 0.05) were selected (**Figure 7A**). Particularly, 28 and 39 KO-associated pathways were identified as COVID-19– and CTRL-specific features, respectively (**Table S5**). Among the others, lipopolysaccharide and peptidoglycan biosynthesis (ko00540 and ko00550, respectively); biosynthesis of amino acids (ko01230); cysteine and methionine metabolism (ko00270); plant–pathogen interaction (ko04626); and vitamin B6 metabolism (ko00750) resulted as specifically associated to patients with COVID-19; whereas ether lipid metabolism (ko00565); fatty acid degradation (ko00071) and valine, leucine, and isoleucine degradation (ko00280) to CTRL subjects.

Both PCA and PLS interpretable visualizations, based on of KO feature projection, clearly identified two separated COVID-19– and CTRLs-related clusters (**Figures 7B, C**).

Remarkably, ML model for GM functional biomarker prediction in patients with COVID-19, compared with CTRLs identified the following KO markers of disease: K03201, type IV secretion system protein VirB6; K01947, Pantothenate and CoA biosynthesis; and K09929, uncharacterized protein. Moreover, a KO marker of CTRL resulted as K18941, two-component system, OmpR family, and response regulator ArlR negatively associated to *Faecalibacterium* spp. (<0.05) and positively associated do *Bifidobacterium* (p < 0.001) (**Figures S15**, **S16**).

### Correlation Between COVID-19 GM and Host Inflammatory and Immunological Markers

To study the bidirectional influence between inflammatory blood proteins levels and the gut ASV abundance in patients with COVID-19, we performed a correlation analysis at L2, L5, and L6, stratifying the patients for disease severity.

At L2, a statistically significant positive correlation (FDR < 0.05) between Bacteroidetes and pro-inflammatory cytokines (e.g., TNFB and ITGA11) was found in both “mild” and “asymptomatic” disease groups; however, focusing onto each correlation pattern between cytokines and Bacteroidetes, DNER, DPP10, PTH1R, TRANCE, and TNFR9 were positively correlated only with the “mild” group, whereas no correlation was found for the “asymptomatic” one. Looking at L5, Bacteroidaceae positively correlated (FDR < 0.05) with ITGA11, CLECA4, and DNER in both “mild” and “asymptomatic” groups. At L6 level, for *Bacteroides*, a statistically significant positive correlation (FDR < 0.05) with DNER, ITGA11, and CLECA4 proteins was observed, regardless of disease severity. On the contrary, a statistically significant negative correlation (FDR < 0.05) was observed for *Sutterella* and 12 proteins (CXCL1, CCL20, DCTN1, CXCL5, GLB1, ZBTB16, PRDX3, TGF-B1, CXCL1, MMP-1, AXIN1, and CXCL10), mostly involved in enhancement of inflammatory processes, for both “mild” and “asymptomatic” disease groups, as well as for the for the entire COVID-19 cohort. The “moderate” subgroup was excluded because of the narrowest of sample number (**Figure S17**).

## Discussion

To our knowledge, this is the first study describing the GM of children affected by SARS-CoV-2 infection.

The GM ecology of children with COVID-19 resulted as dysbiotic, characterized by the lowest α-diversity compared with Non–COVID-19 and CTRLs, a specific β-diversity, a global ASV distribution rich in Fusobacteria and Bacteroidetes, and poor in Actinobacteria and Verrucomicrobia, mainly reflecting an increase of opportunistic pathogens and a depletion of beneficial commensal.

Similar results were obtained by Zuo et al., who described a microbiota profile in adult patients affected by COVID-19 enriched in *Clostridium hathewayi*, *Actinomyces viscosus*, and *Bacteroides nordii* compared with the negative COVID-19 subjects (Zuo et al., 2020). According to that, Gu et al. found a significantly reduced bacterial diversity, a significantly higher relative abundance of opportunistic pathogens, such as *Streptococcus*, *Rothia*, *Veillonella*, and *Actinomyces*, and a lower relative abundance of beneficial symbionts in adult patient with SARS-CoV-2 infection (Gu et al., 2020).

Studies have shown that respiratory viral infection can alter the GM in children such as respiratory syncytial virus (Harding et al., 2020) and H1N1 influenza A virus (Hanada et al., 2018). The interplay between the GM, invasive viruses, and host physiology is complex and yet to be fully characterized, but increasingly, the evidence shows that the microbiome can have an impact on viral disease outcomes (Harper et al., 2020).

In our study, the GM of children with SARS-CoV-2 infection was significantly poor of commensal such as *Akkermansia*, *Blautia*, and *Ruminococcus* compared with healthy subjects. In detail, *Akkermansia*, belonging to the Verrucomicrobia phylum, has an essential role in maintaining the intestinal homeostasis due to a strong interplay with both the host cells and the gut microbial community, and it is crucial for guaranteeing a proper mucus production and thickness (Lopetuso et al., 2020). *Akkermansia* decline has been proposed to have a crucial role in affecting microbial ecology and in discriminating patient from healthy subject as a strong marker of dysbiosis (Lopetuso et al., 2020). *Blautia* species depletion has been correlated in the literature with dysbiosis status in obese pediatric patient with a proinflammatory response (Benítez-Páez et al., 2020), whereas a reduction of *Ruminococcus* species has been reported in inflammatory bowel diseases (IBDs) (Schirmer et al., 2019). Together, these results highlighted that SARS-Cov-2 can affect the GM reducing some of the shortest-chain fatty acids (SCFAs) producing commensal.

As previous finding in adult patients, in our study, GM of patients with COVID-19 showed elevated levels of *Rothia* (Gu et al., 2020; Wu et al., 2021). Indeed, Marotz et al., testing whether specific bacterial communities may predict SARS-CoV-2 occurrence in a hospital setting, found that *Rothia* strongly predicted SARS-CoV-2 presence, with greater prevalence in SARS-CoV-2–positive surface and human samples, even when compared with samples from patients in other intensive care units prior to the COVID-19 pandemic (Marotz et al., 2021).

Remarkably, we found an increase of *Fusobacterium* in the GM of children affected by SARS-CoV-2 infection. Interestingly, this species has been associated with the pathogenicity of inflammatory disorder, including IBDs, acute appendicitis, and colorectal cancer, suggesting a role in regulating local gut immunity inducing a systemic immune activation (Lee et al., 2018). Tao et al. reported that in adult patient with COVID-19, fecal IL-18 levels positively correlated with the relative abundance of *Peptostreptococcus*, *Fusobacterium*, and *Citrobacter*, indicating that changes in GM composition might contribute to SARS-CoV-2–induced production of inflammatory cytokines in the intestine (Tao et al., 2020).

Despite a depletion of *Faecalibacterium* was described by more authors in adult patients with COVID-19 (Gu et al., 2020; Zuo et al., 2020; Yeoh et al., 2021), in our study, we found an increase of *Faecalibacterium* in GM of COVID-19 pediatric patients compared with the healthy subjects. Furthermore, to investigate whether GM profile could be predictive of COVID-19 disease was used, the ML analyses and indeed this analysis revealed that some intestinal bacteria had capability to classify the 90% of the COVID-19 and CTRLs. Remarkably, *Faecalibacterium* showed high important score in the model prediction. Recently, Reinold et al. described the GM of adult patients with severe/critical COVID-19 characterized by a low abundance of butyrate-producing genera such as *Faecalibacterium* and *Roseburia* and identified these two genera as the only able to discriminate severe/critical from non-severe COVID-19 illness (Reinold et al., 2021); in addition, on the basis of this, two microbes that are SARS-CoV-2–negative were distinguished from patients with mild COVID-19 (Reinold et al., 2021).

However, *Faecalibacterium* also functions in preventing IBDs by secreting other anti-inflammatory molecules such as salicylic acid and microbial anti-inflammatory molecule (MAM) (Tian et al., 2021).

Furthermore, the GM profile of patients with COVID-19 appeared unmodified during hospital stay suggesting an immediate GM quenching response to SARS-CoV-2 infection since the first hours of the symptoms’ appearance (Zuo et al., 2020; Yeoh et al., 2021).

Children affected by COVID-19 are often asymptomatic (in 43%–68% of cases) or have mild symptoms, and life-threatening illness and death from COVID-19 are rare (Molteni et al., 2021). Since the pandemic begin, more hypotheses have been postulated: cross-reactive antibodies to common-cold coronaviruses, a stronger innate immune response in children and constitutive differences in immune system states between young and old people, e.g., the skewing of T cells from T helper type 1 (TH1) toward more TH2 in young children (Brodin, 2021; Zimmermann and Curtis, 2022), and differences in oropharyngeal, nasopharyngeal, lung and/or GI microbiota of children compared with the adults (Zimmermann and Curtis, 2021; Zimmermann and Curtis, 2022). As expected, in our cohort, we did not have any severe cases; however, we compared GM profile among “asymptomatic”, “mild”, and “moderate” cases of COVID-19. Unlike the adults (Reinold et al., 2021), we did not find any significant difference among the three groups.

Remarkably, the antibiotic therapy regimen did not significantly impact the α-diversity of GM and on ASV distribution. This result highlighted that GM chances induce by the SARS-CoV-2 were not influenced by the antibiotic administration. Our data differed from what found in the study of Cao et al., where a significant difference in α- and β-diversity was found between adult patients with COVID-19 using antibiotic and those not using antibiotic (Cao et al., 2021).

In our cohort of COVID-19, 52% had SARS-Cov-2 virus detectable on stools; our data disagreed with Rosario et al., who found a reduced α-diversity in the group of stools positive to SARS-CoV-2 (Moreira-Rosário et al., 2021). Nowadays, the active of role of SARS-CoV-2 in gut is still in debate (Guo et al., 2021).

Although the small sample size of MIS-C cohort, its GM was enriched of bacteria considered biomarker of IBDs such as *Fusobacterium* and *Ruminococcus* (Zhou et al., 2021). Although GI symptoms predominate MIS-C symptomatology, the role of the GI tract in the pathogenesis of MIS-C has not been evaluated. Yonker et al. showed that in children with MIS-C, a prolonged presence of SARS-CoV-2 in the GI tract led to the release of zonulin, a biomarker of intestinal permeability, with subsequent trafficking of SARS-CoV-2 antigens into the bloodstream, leading to hyperinflammation (Yonker et al., 2021). As zonulin-dependent loss of gut integrity develops in MIS-C but not COVID-19–infected children, this suggests that a chronicity of SARS-CoV-2 dysbiosis in the gut results in a cumulative increase in the breakdown of mucosal barrier integrity (Yonker et al., 2021). In addition, this suggests that intervening and preventing mucosal epithelial damage early in the disease course could prevent the development of MIS-C (Yonker et al., 2021).

Interestingly, looking at the KO-based prediction of functional profile of microbial communities of COVID-19 and healthy subjects, we identified 28 and 39 specific KO-associated pathways, respectively.

In our study, we found an association between the lipopolysaccharide biosynthesis and the COVID-cohort; therefore, in detail, our results provide an interesting molecular link between inflammation during SARS-CoV-2 infection and comorbidities involving increased levels of bacterial endotoxins (Petruk et al., 2020). However, in our cohort, we also observed an overexpression of peptidoglycan biosynthesis, probably associated to *Bacteroides* increase in our microbiota profile*. Bacteroides* is recognized in the literature as quencher agent of inflammation in IBDs (Jones et al., 2020).

Generally, for adult patients with COVID-19, the metabolisms of main amino acids, nucleotides, organic acids, and carbohydrates are significantly decreased (Soeters and Grecu, 2012; Wu et al., 2020).

Wu et al., in adult fatal cases of COVID-19, described a downregulation of carbamoyl phosphate synthesis with a consequent impairment of urea cycle and removal of excess ammonia, suggesting the possibility of liver damage and therefore an impairment of liver immune function (Wu et al., 2020). Interestingly, in our pediatric cohort, we found an overexpression of amino acid biosynthesis in COVID-19, suggesting an adequate liver function and, consequently, effective immunological response (Soeters and Grecu, 2012).

Moreover, in our COVID-19 cohort, the KO function plant–pathogen interaction was associated with the disease and not with the CTRLs. This pathway is related to the plants lack animal-like adaptive immunity mechanisms, and therefore, it is involved in a specific system with multiple layers against invading pathogens (https://www.genome.jp/dbget-bin/www_bget?ko04626).

The same pathway resulted as correlated to Fusobacteria and Leptotrichia in non-HPV head and neck squamous cell carcinoma microbiome (Chen et al., 2020b). Despite that we did not find the same tight correlation with Fusobacteria, it is noteworthy that this phylum was significantly representative of our COVID-19 cohort. In our cohort, the lipids involved in glycerol metabolism pathway were upregulated in CTRLs; recent data showed that the same pathways upregulated in adult patients with severe COVID-19 (Wu et al., 2020). These energy metabolites could be expended for viral replication, suggesting that SARS-CoV-2 probably hijacks cellular metabolism like many other viruses (Thaker et al., 2019). In the CTRLs, but not in pediatric COVID-19 cohort, we observed an upregulation of this pathway.

Furthermore, our functional study revealed the fatty acid degradation pathway significantly enriched in CTRLs and not in COVID-19 cohort. The same pathway is described in the literature in correlation with an inflammatory condition as reported by Manor et al., where the microbiome of patient with cystic fibrosis (Manor et al., 2016) is characterized by an enrichment of fatty acid degradation pathway compared with CTRLs and by a decreased capacity for overall fatty acid biosynthesis (Manor et al., 2016). For the last function, we did not observe any difference between COVID-19 and CTRLs.

About the function of fatty acid metabolism, this resulted to be overexpressed in our CTRLs. The fatty acid metabolism is crucial for an adequate immune response (Batista-Gonzalez et al., 2020), especially for the macrophage activation. An altered lipid metabolism in morbidly obese individuals is described to affect the immune response and the disease severity in adult patients with COVID-19 (Tanner and Alfieri, 2021).

These data support the hypothesis that the microbiome in pediatric COVID-19 seems to be different from adult patients with COVID-19, and, actually, it contributes with its anti-inflammatory properties to reduce the disease severity.

L-isoleucine is a crucial mediator in the microbiota–host cross-talk, and it plays an important role in regulating host innate and adaptive immunity (Gu et al., 2019). Zhang et al. described, in adult patients with severe COVID-19, a depletion of beneficial microbial functions characterized by impairment of carbohydrate degradation, capacity for SCFA production, and L-isoleucine biosynthesis (Zhang et al., 2022). In our study, we found an absence of L-isoleucine degradation function in COVID-19 cohort, whereas this function resulted as specifically associated to the CTRLs. Moreover, in the same study, Zhang et al. described a microbiota of severe COVID-19 depleted of *Faecalibacterium* (Zhang et al., 2022); it is noteworthy that, in our study, this genus was significantly correlated with our pediatric COVID-19. As known, this bacterium exerts a crucial role in maintaining the gut homeostasis, because of its anti-inflammatory and fermentative properties (e.g., SCFA production). Remarkably, ML model for GM microbial biomarker prediction identified *Faecalibacterium* as a feature of COVID-19.

GM ecology and correlation analysis against blood inflammatory proteins, related to disease severity, confirmed the proinflammatory role of *Bacteroides*, although dampened by a peptidoglycan biosynthesis pathway, specific of the COVID-19 subgroup. Moreover, the negative correlation of *Sutterella* with 13 proinflammatory cytokines corroborates the evidence that this microorganism is a widely prevalent intestinal commensal with mild pro-inflammatory capacity. In fact, *Sutterella* is able to adhere to intestinal epithelial cells, indicating its possible role in immunomodulation of intestine, but it lacks of the capability to disrupt the gut homeostasis (Hiippala et al., 2016).

One caveat of this study was the poor samples size of stool samples collected at T2 and the absence of samples collected during a follow-up, limiting the analyses of SARS-CoV-2 long-term effect on GM in children. We also had a limited number of patients affected by MIS-C, which affected the understanding of the actual differences between children with MIS-C and healthy controls. Moreover, patients included in the COVID-19 and Non–COVID-19 cohorts received antibiotic therapy during the time of the study, which can be a confounding factor for GM profiling.

## Conclusion

Together, these data support the hypothesis that the GM in pediatric patients with COVID-19, unlike in the adult, contributes with its anti-inflammatory properties to reduce or avoid a severe disease. This highlights a potential link between GM functions and clinical course of COVID-19 in pediatric patients, playing a central role in deciphering the low severity disease of children to SARS-CoV-2 infection. Our study may shed light to investigate the potential role of pediatric GM to ameliorate the disease in adult.

## Consortium

CACTUS Study Team: Francesca Calo` Carducci, MD, Caterina Cancrini, MD, PhD, Sara Chiurchiù, MD, Marta Ciofi degli Atti, MD, Laura Cursi, MD, Renato Cutrera, MD, Carmen D’Amore, MD, Patrizia D’Argenio, MD, Maria A. De Ioris, MD, Maia De Luca, MD, Andrea Finocchi, MD, PhD, Laura Lancella, MD, Emma Concetta Manno MD, Elena Morrocchi, PhD, Paola Pansa, MD, Libera Sessa, PhD, Paola Zangari, MD.

## Data Availability Statement

The datasets presented in this study can be found in online repositories. The names of the repository/repositories and accession number(s) can be found below: https://www.ncbi.nlm.nih.gov/, PRJNA753792.

## Ethics Statement

The studies involving human participants were reviewed and approved by Bambino Gesù Children’s Hospital Ethical Commettee. Written informed consent to participate in this study was provided by the participants’ legal guardian/next of kin.

## Author Contributions

LP and LR conceived the study and wrote the manuscript. GM, VG, SG, and GP analyzed microbiota profiling data. FDC revised microbiota profiling analysis and manuscript content. MVR and SP managed sample manipulation and related dataset. MVR performed sequencing. NC reviewed the immunological data. SB, AC, LR, and PP managed the patients with COVID-19. AV, PR, and CFP reviewed the manuscript. All the authors reviewed the final version of the manuscript and agreed to be accountable for the content of the work.

## Funding

The study was funded by Ministry of Health (Ricerca Corrente RC2021_Modale_Putignani; 202005_Genetica_Putignani) and by “The BioArte Limited” Company from Malta (202003_Bioarte_Putignani) to LP.

## Conflict of Interest

Author GM, VG, and SG are employed by GenomeUp SRL, Viale Pasteur, 6, 00144, Rome, Italy.

The remaining authors declare that the research was conducted in the absence of any commercial or financial relationships that could be construed as a potential conflict of interest.

## Publisher’s Note

All claims expressed in this article are solely those of the authors and do not necessarily represent those of their affiliated organizations, or those of the publisher, the editors and the reviewers. Any product that may be evaluated in this article, or claim that may be made by its manufacturer, is not guaranteed or endorsed by the publisher.
