# Supplementary material for: The Relationship Between Pediatric Gut Microbiota and SARS-CoV-2 Infection
Source: Front Cell Infect Microbiol. 2022 Jul 8;12:908492. doi: 10.3389/fcimb.2022.908492 (PMC9304937; doi:10.3389/fcimb.2022.908492)
Supplement: Supplementary file 1 [file DataSheet_1.docx]

Supplementary Material

Tables

**Table S1. Study cohorts and stool samples collected.**

| **Cohort** | **Number of stool samples** |
| --- | --- |
| COVID-19 (T_0_) | 68 |
| COVID-19 (T_1_) | 27 |
| COVID-19 (T_2_) | 11 |
| Non COVID-19 (T_0_) | 16 |
| Non COVID-19 (T_1_) | 6 |
| Non COVID-19 (T_2_) | 1 |
| MIS-C (T_0_) | 4 |
| MIS-C (T_1_) | 2 |
| MIS-C (T_2_) | 1 |
| CTRLs | 95 |

**Table S2. Reads number for each sample.**

| **sample-id** | **Category** | **#input reads** | **sample-id** | **Category** | **#input reads** | **sample-id** | **Category** | **#input reads** | **sample-id** | **Category** | **#input reads** |
| --- | --- | --- | --- | --- | --- | --- | --- | --- | --- | --- | --- |
| C527 | COVID-19 | 148479 | C341 | COVID-19 | 16489 | C300 | NO COVID | 31020 | N-11-3 | CTRL | 47148 |
| C485 | COVID-19 | 108569 | C85 | COVID-19 | 14638 | C51 | NO COVID | 21468 | N-03-6 | CTRL | 46756 |
| C491 | COVID-19 | 102045 | C63 | COVID-19 | 14525 | C103 | NO COVID | 12386 | N-99-2 | CTRL | 45956 |
| C449 | COVID-19 | 92809 | C243 | COVID-19 | 13930 | C362 | NO COVID | 12358 | N-08-6 | CTRL | 45276 |
| C509 | COVID-19 | 86716 | C346 | COVID-19 | 11295 | C366 | NO COVID | 10072 | N-11-11 | CTRL | 44625 |
| C144 | COVID-19 | 84971 | C395 | COVID-19 | 10168 | C406 | NO COVID | 6233 | N-00-3 | CTRL | 44023 |
| C510 | COVID-19 | 83032 | C519 | COVID-19 | 7426 | C72 | NO COVID | 6206 | N-06-2 | CTRL | 43269 |
| C507 | COVID-19 | 79748 | C2 | COVID-19 | 6410 | C520 | NO COVID | 1624 | N-11-8 | CTRL | 42787 |
| C269 | COVID-19 | 77167 | C168 | COVID-19 | 5847 | C203 | NO COVID | 1306 | N-05-3 | CTRL | 42631 |
| C154 | COVID-19 | 76830 | C231 | COVID-19 | 4097 | N-00-5 | CTRL | 145824 | N-10-7 | CTRL | 41102 |
| C36 | COVID-19 | 76331 | C94 | COVID-19 | 3148 | N-03-4 | CTRL | 143106 | N-09-10 | CTRL | 40198 |
| C159 | COVID-19 | 74385 | C303 | COVID-19 | 2923 | N-97-1 | CTRL | 134845 | N-05-1 | CTRL | 40090 |
| C87 | COVID-19 | 73280 | C170 | COVID-19 | 2883 | N-02-7 | CTRL | 129263 | N-11-7 | CTRL | 39034 |
| C114 | COVID-19 | 73082 | C66 | COVID-19 | 2742 | N-02-4 | CTRL | 122084 | N-04-2 | CTRL | 38683 |
| C336 | COVID-19 | 72219 | C174 | COVID-19 | 2627 | N-00-2 | CTRL | 121177 | N-96-1 | CTRL | 38641 |
| C133 | COVID-19 | 68718 | C205 | COVID-19 | 2582 | N-04-1 | CTRL | 120872 | N-07-6 | CTRL | 38443 |
| C128 | COVID-19 | 65611 | C425 | COVID-19 | 2090 | N-00-4 | CTRL | 118160 | N-10-8 | CTRL | 37592 |
| C445 | COVID-19 | 65113 | C121 | COVID-19 | 2082 | N-05-4 | CTRL | 110696 | N-05-2 | CTRL | 37524 |
| C13 | COVID-19 | 64540 | C472 | COVID-19 | 2050 | N-08-3 | CTRL | 99646 | N-07-5 | CTRL | 37351 |
| C321 | COVID-19 | 63445 | C225 | COVID-19 | 1618 | N-08-1 | CTRL | 87915 | N-11-5 | CTRL | 36845 |
| C228 | COVID-19 | 63351 | C358 | COVID-19 | 1538 | N-11-6 | CTRL | 85570 | N-12-1 | CTRL | 36104 |
| C246 | COVID-19 | 62441 | C467 | COVID-19 | 1326 | N-03-5 | CTRL | 85198 | N-11-4 | CTRL | 35910 |
| C293 | COVID-19 | 59564 | C213 | COVID-19 | 1298 | N-11-2 | CTRL | 82807 | N-98-3 | CTRL | 35594 |
| C515 | COVID-19 | 58985 | C193 | COVID-19 | 1042 | N-01-2 | CTRL | 82277 | N-06-6 | CTRL | 35253 |
| C273 | COVID-19 | 57771 | C416 | COVID-19++ | 74945 | N-04-6 | CTRL | 77257 | N-10-1 | CTRL | 33362 |
| C39 | COVID-19 | 57008 | C422 | COVID-19++ | 72096 | N-02-2 | CTRL | 62735 | N-07-3 | CTRL | 29584 |
| C389 | COVID-19 | 56148 | C512 | COVID-19++ | 51582 | N-05-9 | CTRL | 62059 | N-98-1 | CTRL | 29468 |
| C234 | COVID-19 | 55871 | C513 | COVID-19++ | 40401 | N-02-1 | CTRL | 60534 | N-05-8 | CTRL | 27677 |
| C481 | COVID-19 | 55024 | C381 | COVID-19++ | 35340 | N-06-4 | CTRL | 60402 | N-10-4 | CTRL | 26874 |
| C493 | COVID-19 | 52302 | C412 | COVID-19++ | 31949 | N-99-1 | CTRL | 60173 | N-05-7 | CTRL | 26208 |
| C217 | COVID-19 | 51349 | C379 | COVID-19++ | 31343 | N-06-5 | CTRL | 58427 | N-00-6 | CTRL | 25280 |
| C511 | COVID-19 | 48657 | C409 | COVID-19++ | 29361 | N-07-12 | CTRL | 58378 | N-11-10 | CTRL | 24387 |
| C118 | COVID-19 | 47434 | C421 | COVID-19++ | 15459 | N-04-3 | CTRL | 57276 | N-09-9 | CTRL | 23403 |
| C263 | COVID-19 | 46987 | C23 | COVID-19++ | 8747 | N-03-8 | CTRL | 57024 | N-09-4 | CTRL | 22859 |
| C502 | COVID-19 | 45427 | C186 | MIS-C | 106011 | N-03-2 | CTRL | 56550 | N-07-4 | CTRL | 21908 |
| C24 | COVID-19 | 44783 | C251 | MIS-C | 59845 | N-03-7 | CTRL | 56405 | N-06-8 | CTRL | 21759 |
| C111 | COVID-19 | 42973 | C508 | MIS-C | 44347 | N-05-5 | CTRL | 56061 | N-04-4 | CTRL | 19555 |
| C17 | COVID-19 | 41598 | C184 | MIS-C | 28714 | N-06-1 | CTRL | 55559 | N-10-6 | CTRL | 19400 |
| C12 | COVID-19 | 37476 | C182 | MIS-C | 2679 | N-11-13 | CTRL | 54259 | N-09-7 | CTRL | 18889 |
| C26 | COVID-19 | 35748 | C283 | NO COVID | 65444 | N-03-1 | CTRL | 52099 | N-98-2 | CTRL | 15615 |
| C19 | COVID-19 | 34536 | C48 | NO COVID | 59856 | N-12-2 | CTRL | 49995 | N-10-5 | CTRL | 14213 |
| C352 | COVID-19 | 31187 | C332 | NO COVID | 58544 | N-02-3 | CTRL | 49355 | N-04-8 | CTRL | 13381 |
| C291 | COVID-19 | 31045 | C278 | NO COVID | 50861 | N-12-3 | CTRL | 49095 | N-04-5 | CTRL | 12852 |
| C288 | COVID-19 | 30728 | C329 | NO COVID | 50320 | N-02-8 | CTRL | 48909 | N-09-8 | CTRL | 11890 |
| C434 | COVID-19 | 29271 | C138 | NO COVID | 47162 | N-01-1 | CTRL | 48791 | N-00-1 | CTRL | 11378 |
| C516 | COVID-19 | 27323 | C327 | NO COVID | 41201 | N-11-12 | CTRL | 48721 | N-11-1 | CTRL | 10854 |
| C240 | COVID-19 | 22178 | C106 | NO COVID | 40602 | N-99-3 | CTRL | 48283 | N-02-5 | CTRL | 10326 |
| C349 | COVID-19 | 19772 | C137 | NO COVID | 35867 | N-02-6 | CTRL | 48194 | N-11-9 | CTRL | 10064 |
| C529 | COVID-19 | 18409 | C388 | NO COVID | 34179 | N-10-3 | CTRL | 47883 | N-06-7 | CTRL | 9662 |
| C60 | COVID-19 | 16872 | C70 | NO COVID | 33320 | N-08-5 | CTRL | 47719 | N-10-2 | CTRL | 3537 |

**Table S3. Kruskal Wallis test results on taxa relative abundances at L2, L5, L6 taxonomy levels. Please see excel file.**

**Table S4. Classification models applied to discriminate COVID-19 and CTRLs patients on GM composition at genus level.**

| **Model** | **Score** | **Score COVID-19** | **Score ctrl** |
| --- | --- | --- | --- |
| DummyClassifier | 0.5 | 0.0 | 1.0 |
| LogisticRegression | 0.9 | 0.9 | 1.0 |
| SGDClassifier | 0.8 | 0.8 | 0.9 |
| LogisticRegressionCV | 0.9 | 0.8 | 1.0 |
| HistGradientBoostingClassifier | 0.9 | 0.9 | 0.9 |
| RandomForestClassifier | 0.9 | 0.8 | 1.0 |
| ExtraTreesClassifier | 0.8 | 0.7 | 1.0 |
| GradientBoostingClassifier | 0.9 | 0.8 | 0.9 |
| BaggingClassifier | 0.8 | 0.8 | 0.9 |
| AdaBoostClassifier | 0.9 | 0.9 | 0.9 |
| MLPClassifier | 0.9 | 0.8 | 0.9 |
| LinearSVC | 0.9 | 0.8 | 0.9 |
| SVC | 0.8 | 0.6 | 1.0 |
| GaussianNB | 0.6 | 0.4 | 0.9 |
| DecisionTreeClassifier | 0.9 | 0.8 | 0.9 |
| QuadraticDiscriminantAnalysis | 0.5 | 0.0 | 1.0 |
| KNeighborsClassifier | 0.8 | 0.8 | 0.9 |
| GaussianProcessClassifier | 0.8 | 0.6 | 0.9 |

**Table S5.** KEGG orthology in up or low expressed in COVID-19 and CTRLs cohorts.

| KO NODE PATHWAY (ko) | COVID-19 | CTRLS | TRENDS IN COVID | KO NODE PATHWAY (ko) | CTRLs | COVID-19 | TRENDS IN CTRLs |
| --- | --- | --- | --- | --- | --- | --- | --- |
| ko01250 Biosynthesis of nucleotide sugars | 2 | 0 | UP (SPECIFIC) | ko00500 Starch and sucrose metabolism | 4 | 0 | DOWN (SPECIFIC) |
| ko03430 Mismatch repair | 2 | 0 | UP (SPECIFIC) | ko00190 Oxidative phosphorylation | 4 | 0 | DOWN (SPECIFIC) |
| ko05111 Biofilm formation - Vibrio cholerae | 1 | 0 | UP (SPECIFIC) | ko00230 Purine metabolism | 3 | 0 | DOWN (SPECIFIC) |
| ko01230 Biosynthesis of amino acids | 9 | 0 | UP (SPECIFIC) | ko00380 Tryptophan metabolism | 3 | 0 | DOWN (SPECIFIC) |
| ko02010 ABC transporters | 8 | 0 | UP (SPECIFIC) | ko00340 Histidine metabolism | 3 | 0 | DOWN (SPECIFIC) |
| ko00270 Cysteine and methionine metabolism | 5 | 0 | UP (SPECIFIC) | ko00643 Styrene degradation | 2 | 0 | DOWN (SPECIFIC) |
| ko00240 Pyrimidine metabolism | 4 | 0 | UP (SPECIFIC) | ko00360 Phenylalanine metabolism | 2 | 0 | DOWN (SPECIFIC) |
| ko00983 Drug metabolism - other enzymes | 3 | 0 | UP (SPECIFIC) | ko01040 Biosynthesis of unsaturated fatty acids | 2 | 0 | DOWN (SPECIFIC) |
| ko00550 Peptidoglycan biosynthesis | 2 | 0 | UP (SPECIFIC) | ko00220 Arginine biosynthesis | 2 | 0 | DOWN (SPECIFIC) |
| ko02026 Biofilm formation - Escherichia coli | 2 | 0 | UP (SPECIFIC) | ko00720 Carbon fixation pathways in prokaryotes | 2 | 0 | DOWN (SPECIFIC) |
| ko03440 Homologous recombination | 2 | 0 | UP (SPECIFIC) | ko00630 Glyoxylate and dicarboxylate metabolism | 2 | 0 | DOWN (SPECIFIC) |
| ko00470 D-Amino acid metabolism | 2 | 0 | UP (SPECIFIC) | ko00330 Arginine and proline metabolism | 2 | 0 | DOWN (SPECIFIC) |
| ko00710 Carbon fixation in photosynthetic organisms | 2 | 0 | UP (SPECIFIC) | ko00790 Folate biosynthesis | 2 | 0 | DOWN (SPECIFIC) |
| ko03410 Base excision repair | 2 | 0 | UP (SPECIFIC) | ko05200 Pathways in cancer | 1 | 0 | DOWN (SPECIFIC) |
| ko00460 Cyanoamino acid metabolism | 2 | 0 | UP (SPECIFIC) | ko04726 Serotonergic synapse | 1 | 0 | DOWN (SPECIFIC) |
| ko04626 Plant-pathogen interaction | 1 | 0 | UP (SPECIFIC) | ko04936 Alcoholic liver disease | 1 | 0 | DOWN (SPECIFIC) |
| ko00750 Vitamin B6 metabolism | 1 | 0 | UP (SPECIFIC) | ko00982 Drug metabolism - cytochrome P450 | 1 | 0 | DOWN (SPECIFIC) |
| ko05132 Salmonella infection | 1 | 0 | UP (SPECIFIC) | ko04978 Mineral absorption | 1 | 0 | DOWN (SPECIFIC) |
| ko00450 Selenocompound metabolism | 1 | 0 | UP (SPECIFIC) | ko05022 Pathways of neurodegeneration - multiple diseases | 1 | 0 | DOWN (SPECIFIC) |
| ko01051 Biosynthesis of ansamycins | 1 | 0 | UP (SPECIFIC) | ko04141 Protein processing in endoplasmic reticulum | 1 | 0 | DOWN (SPECIFIC) |
| ko05415 Diabetic cardiomyopathy (1) | 1 | 0 | UP (SPECIFIC) | ko00430 Taurine and hypotaurine metabolism | 1 | 0 | DOWN (SPECIFIC) |
| ko05120 Epithelial cell signaling in Helicobacter pylori infection | 1 | 0 | UP (SPECIFIC) | ko04152 AMPK signaling pathway | 1 | 0 | DOWN (SPECIFIC) |
| ko03030 DNA replication | 1 | 0 | UP (SPECIFIC) | ko04216 Ferroptosis | 1 | 0 | DOWN (SPECIFIC) |
| ko00540 Lipopolysaccharide biosynthesis | 1 | 0 | UP (SPECIFIC) | ko00565 Ether lipid metabolism | 1 | 0 | DOWN (SPECIFIC) |
| ko00010 Glycolysis / Gluconeogenesis | 1 | 0 | UP (SPECIFIC) | ko00310 Lysine degradation | 1 | 0 | DOWN (SPECIFIC) |
| ko00970 Aminoacyl-tRNA biosynthesis | 1 | 0 | UP (SPECIFIC) | ko00562 Inositol phosphate metabolism | 1 | 0 | DOWN (SPECIFIC) |
| ko05010 Alzheimer disease | 1 | 0 | UP (SPECIFIC) | ko00071 Fatty acid degradation | 1 | 0 | DOWN (SPECIFIC) |
| ko03420 Nucleotide excision repair | 1 | 0 | UP (SPECIFIC) | ko00910 Nitrogen metabolism | 1 | 0 | DOWN (SPECIFIC) |
| KO NODE PATHWAY (ko) | CTRLs | COVID-19 | TRENDS IN CTRLs | ko05012 Parkinson disease | 1 | 0 | DOWN (SPECIFIC) |
| ko02020 Two-component system | 5 | 0 | DOWN (SPECIFIC) | ko00040 Pentose and glucuronate interconversions | 1 | 0 | DOWN (SPECIFIC) |
| ko00350 Tyrosine metabolism | 5 | 0 | DOWN (SPECIFIC) | ko00909 Sesquiterpenoid and triterpenoid biosynthesis | 1 | 0 | DOWN (SPECIFIC) |
| ko00280 Valine, leucine and isoleucine degradation | 5 | 0 | DOWN (SPECIFIC) | ko05225 Hepatocellular carcinoma | 1 | 0 | DOWN (SPECIFIC) |
| ko00680 Methane metabolism | 4 | 0 | DOWN (SPECIFIC) | ko05208 Chemical carcinogenesis - reactive oxygen species | 1 | 0 | DOWN (SPECIFIC) |
| ko00051 Fructose and mannose metabolism | 4 | 0 | DOWN (SPECIFIC) | ko05134 Legionellosis | 1 | 0 | DOWN (SPECIFIC) |

**Figures**

#
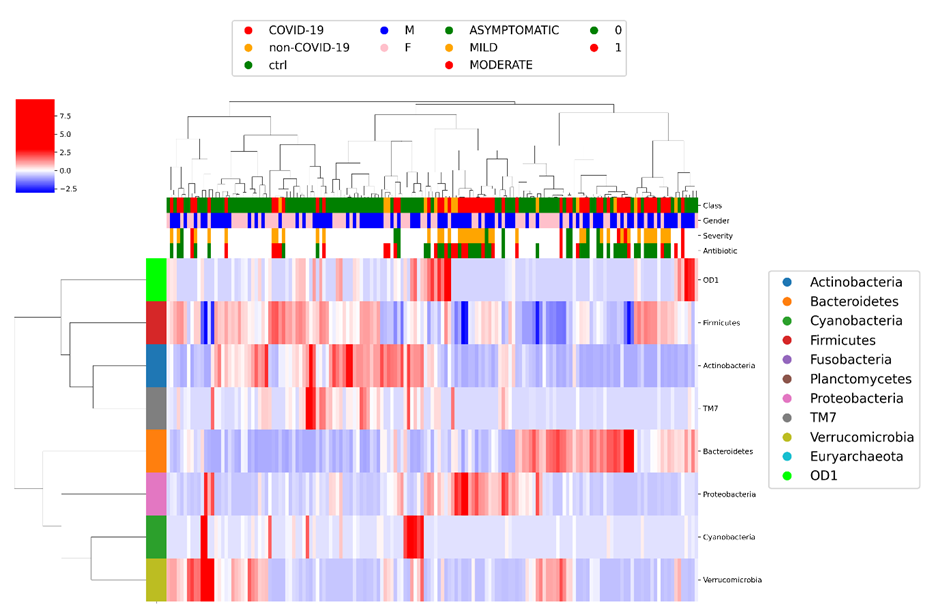


**Figure S1.** Graphical representation of hierarchical analysis of global ASVs distribution at level L2 (phylum) for COVID-19, Non-Covid-19 and CTRLs subjects filtered by a t-test between classes with *p*-value <0.05 at T_0_. In the heatmap, the hierarchical complete linkage dendrogram is based on the ASVs Pearson’s correlation coefficient. The color scale characterizes the Z- score for each variable: red, high level; blue, low level. The column color labels represent respectively: patient’s class (red=COVID-19, orange=Non-COVID-19, green=CTRLs), gender (blue=male, pink=female), severity (green=asymptomatic, orange=mild, red=moderate), and antibiotic (green=absent (0), red=present (1)).


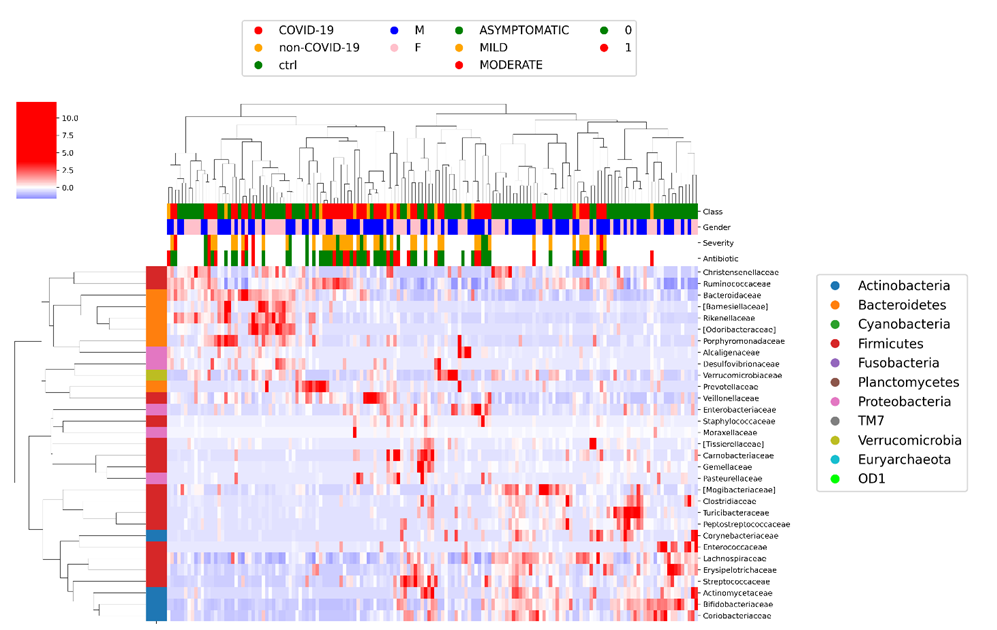


**Figure S2.** Graphical representation of hierarchical analysis of global ASVs distribution at level L5 (family) for COVID-19, Non-Covid-19 and CTRLs subjects filtered by a t-test between classes with *p*-value <0.05 at T_0_. In the heatmap, the hierarchical complete linkage dendrogram is based on the ASVs Pearson’s correlation coefficient. The color scale characterizes the Z- score for each variable: red, high level; blue, low level. The column color labels represent respectively: patient’s class (red=COVID-19, orange=Non-COVID-19, green=CTRLs), gender (blue=male, pink=female), severity (green=asymptomatic, orange=mild, red=moderate), and antibiotic (green=absent (0), red=present (1)).


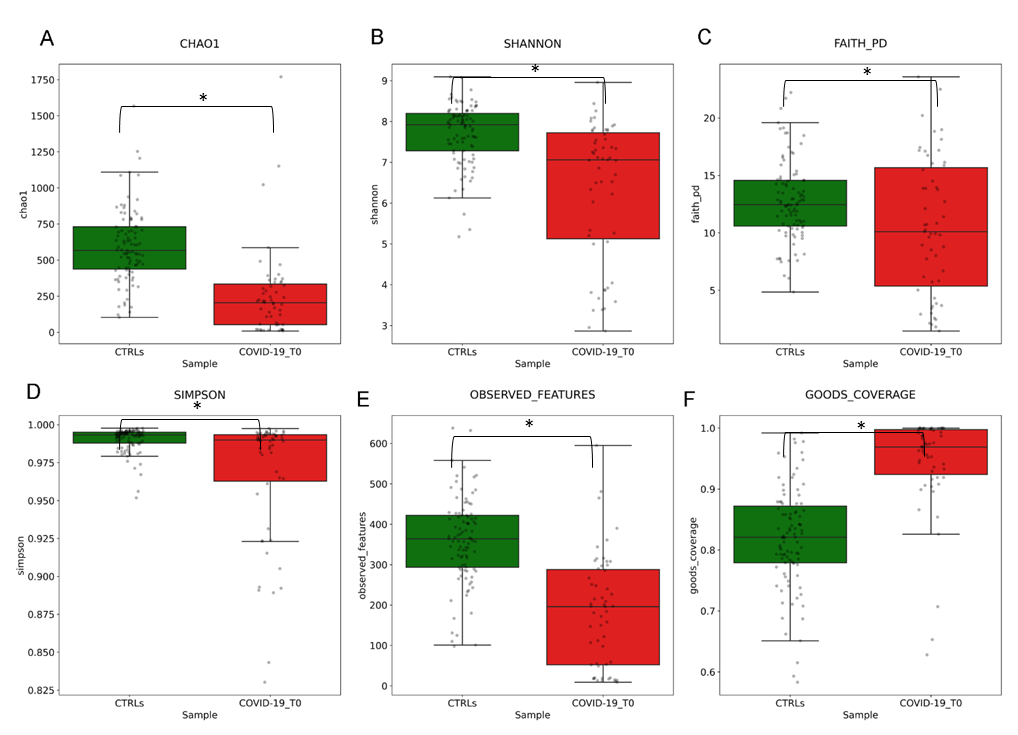


**Figure S3.** Evaluation of the α-diversity among COVID-19 at T_0_ and CTRLs based on Chao-1 (A), Shannon (B), Faith PD (C), Simpson (D), observed features (E), goods coverage (F) indices. Outliers were excluded from plot representation.


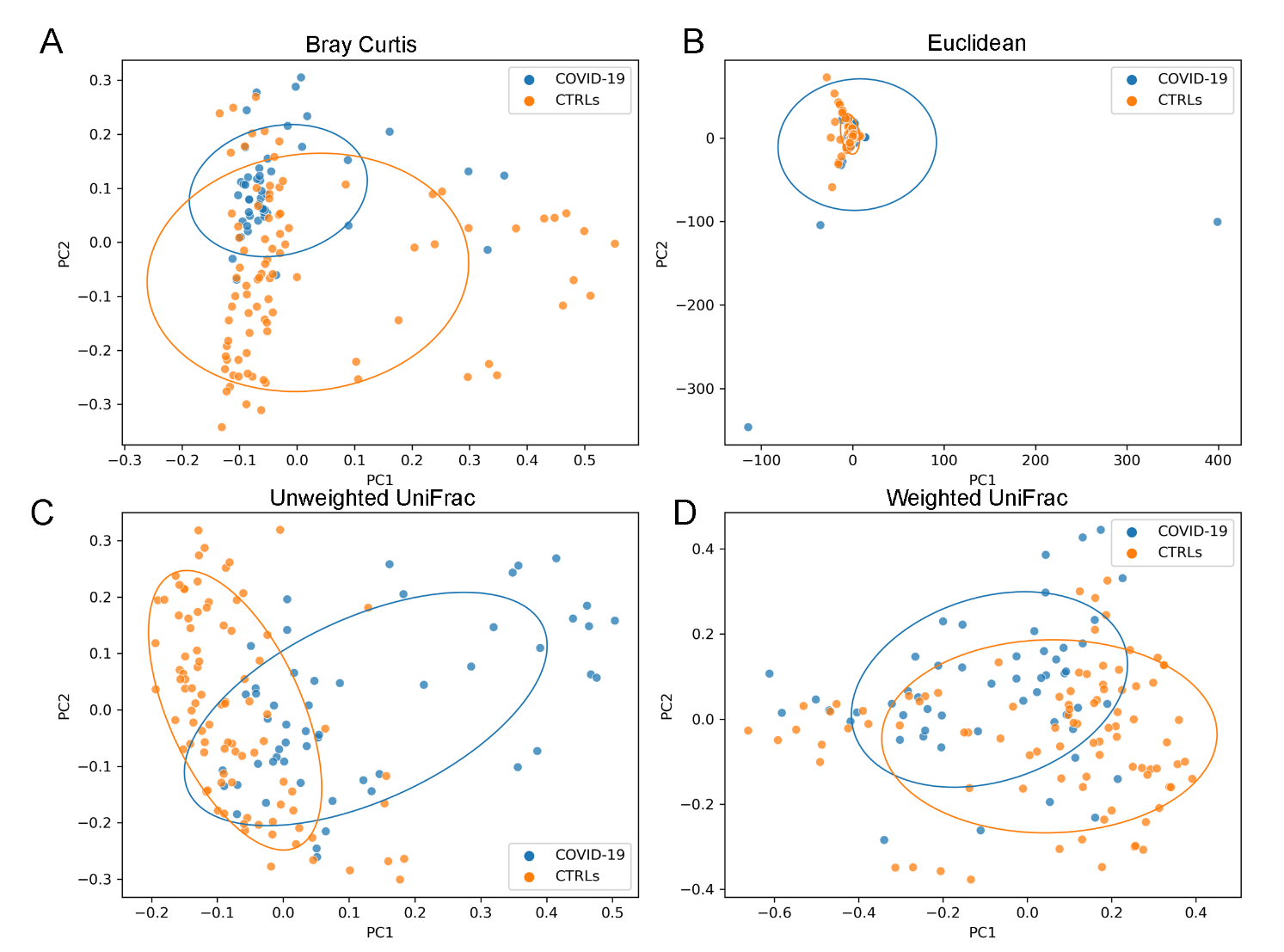


**Figure S4.** Beta-diversity analyses, performed by Bray-Curtis (A), Euclidian distance (B), unweighted (C) and weighted UniFrac (D) algorithms for COVID-19 at T_0_ and CTRLs (PERMANOVA p values < 0.0001).

**
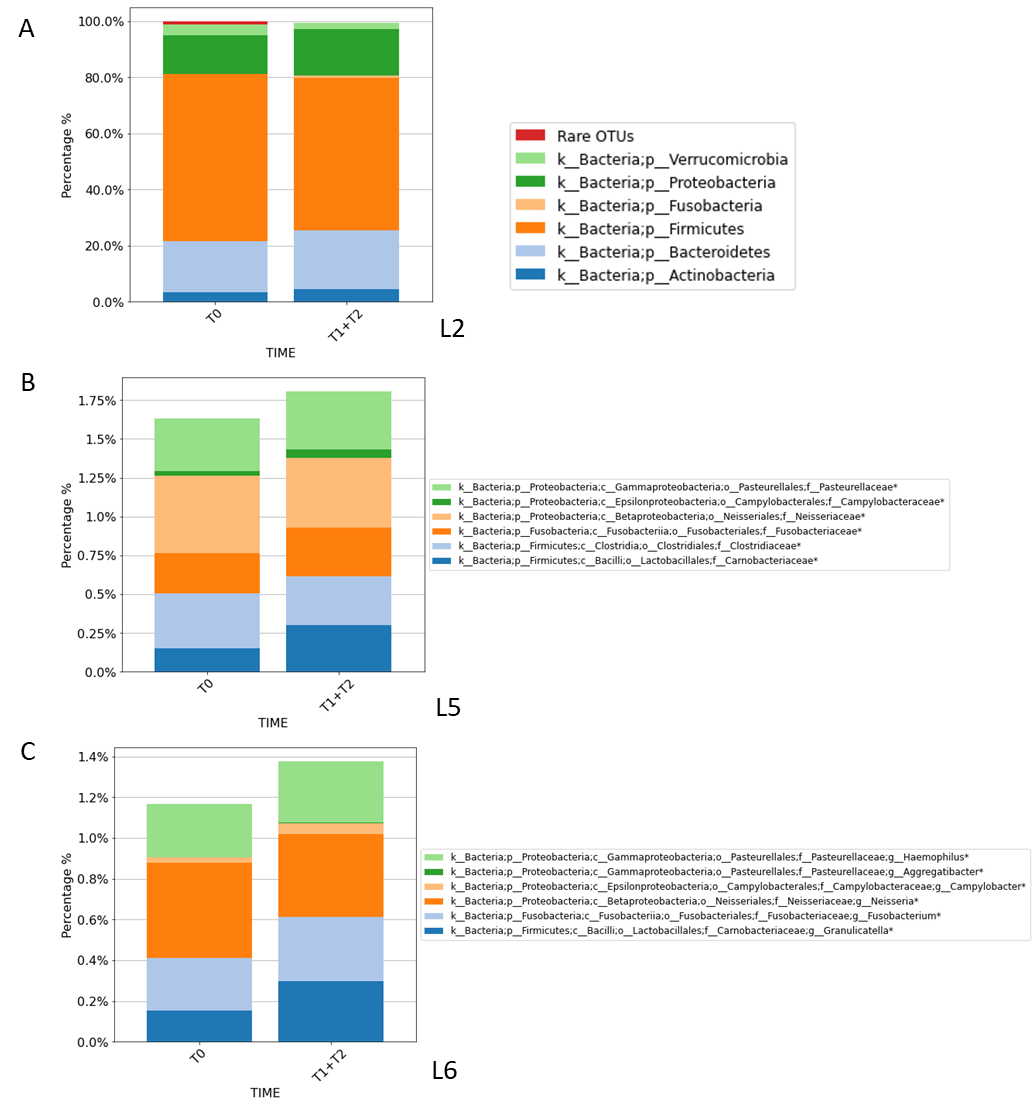
**

**Figure S5** ASV comparison between T_0_, T_1_ and T_2_ at L2 (A), L5 (B), L6 (C) of COVID-19 cohort filtered by statistically significance based on Kruskal-Wallis test. *p value<0.001; p value **FDR<0.05; *** p value FDR<0.001.


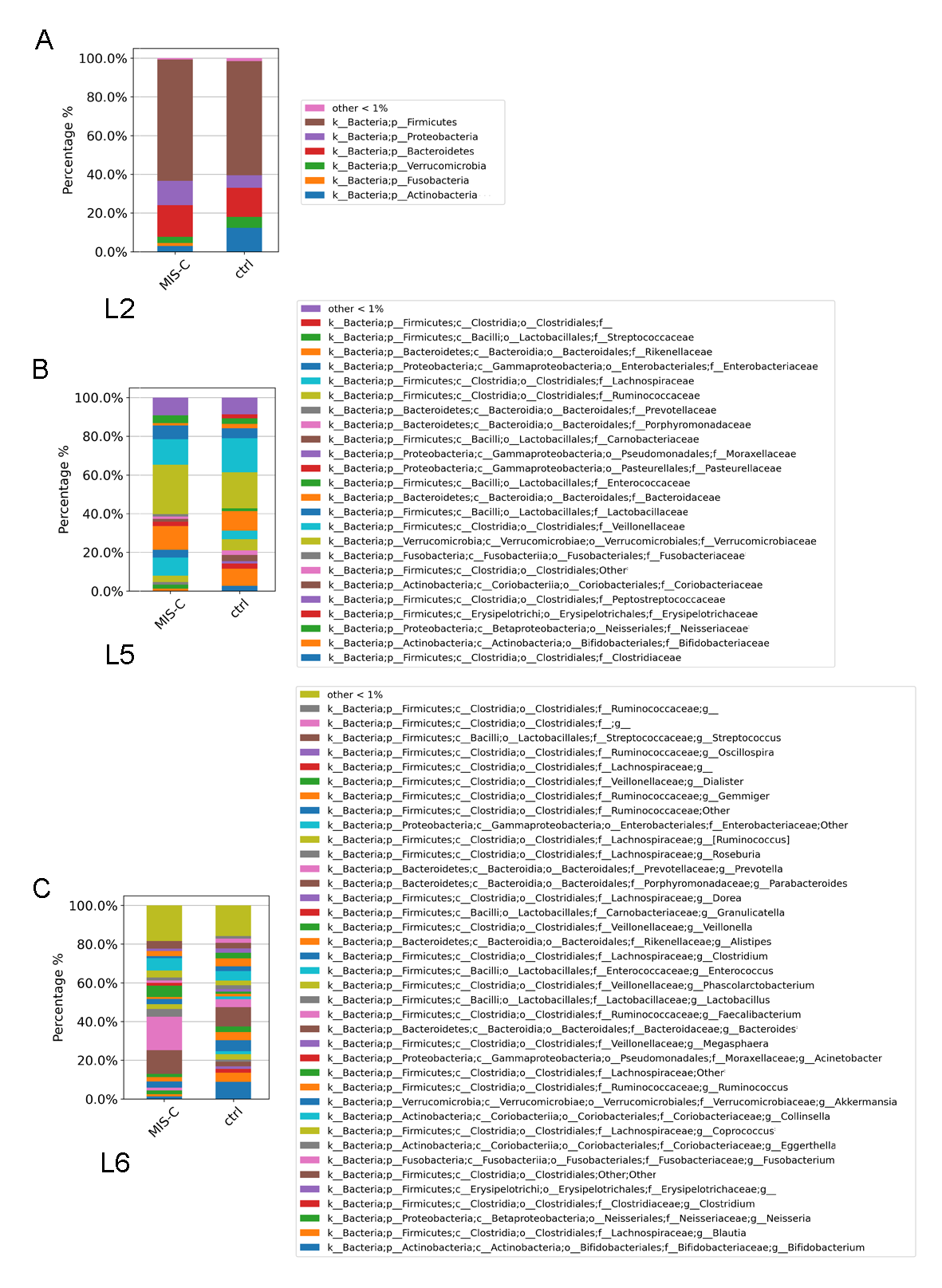


**Figure S6.** ASV distributions at L2, L5, L6 **(Panels A-C)** of MIS-C and CTRL.


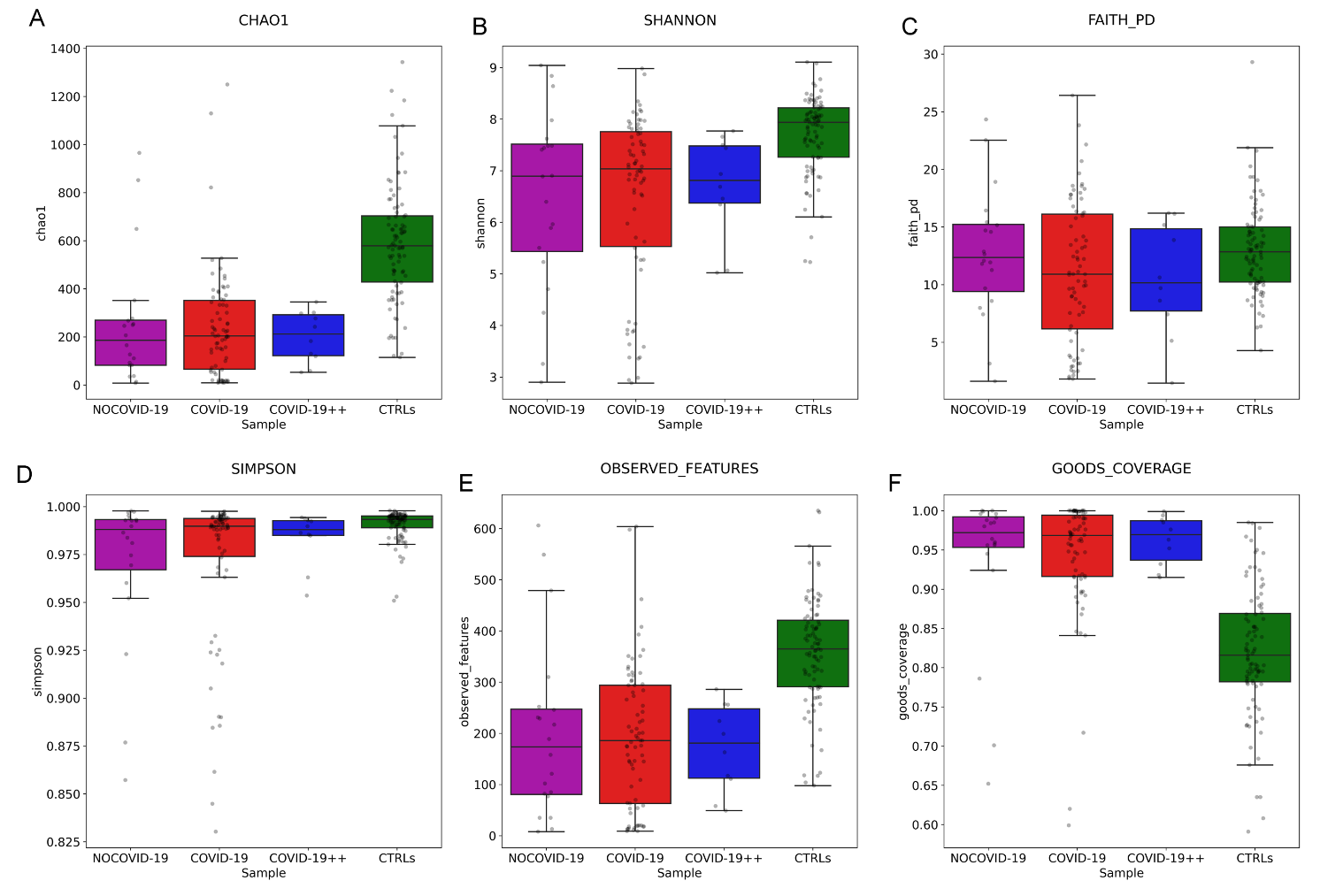


**Figure S7.** Evaluation of the α-diversity among COVID-19, COVID-19^++^, Non Covid-19 cohorts and CTRLs based on Chao-1 (A), Shannon (B), Faith PD (C), Simpson indices (D), Observed Features (E), goods coverage (F) . Outliers were excluded from plot representation.


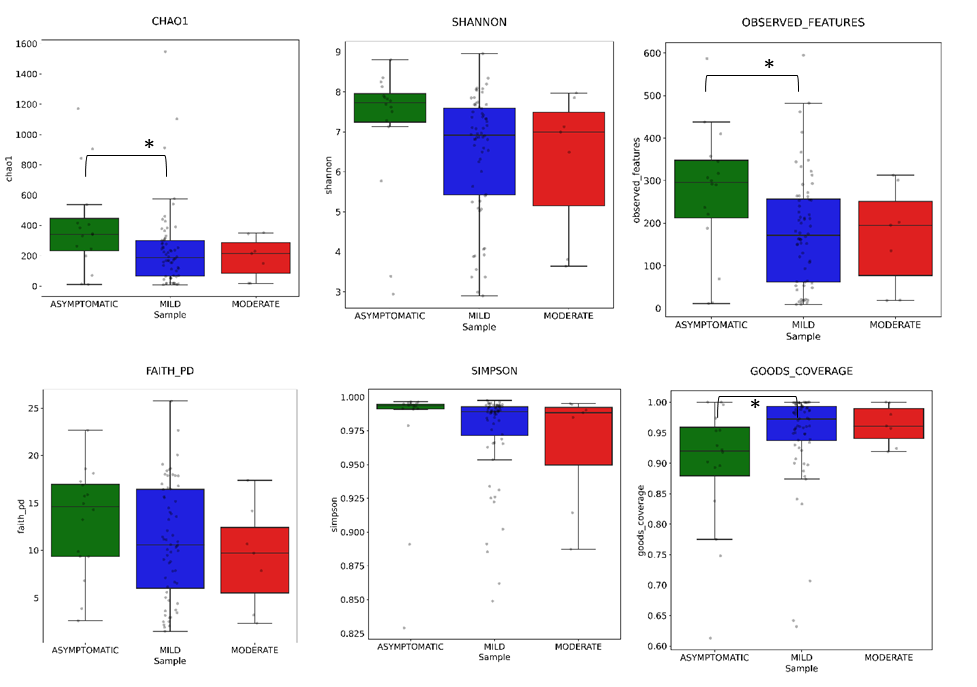


**Figure S8.** Evaluation of the α-diversity among “asymptomatic”, “mild” and “moderate” groups based on Chao-1, Shannon, observed species, phylogenetic distance, goods coverage and Simpson indices. Outliers were excluded from plot representation.


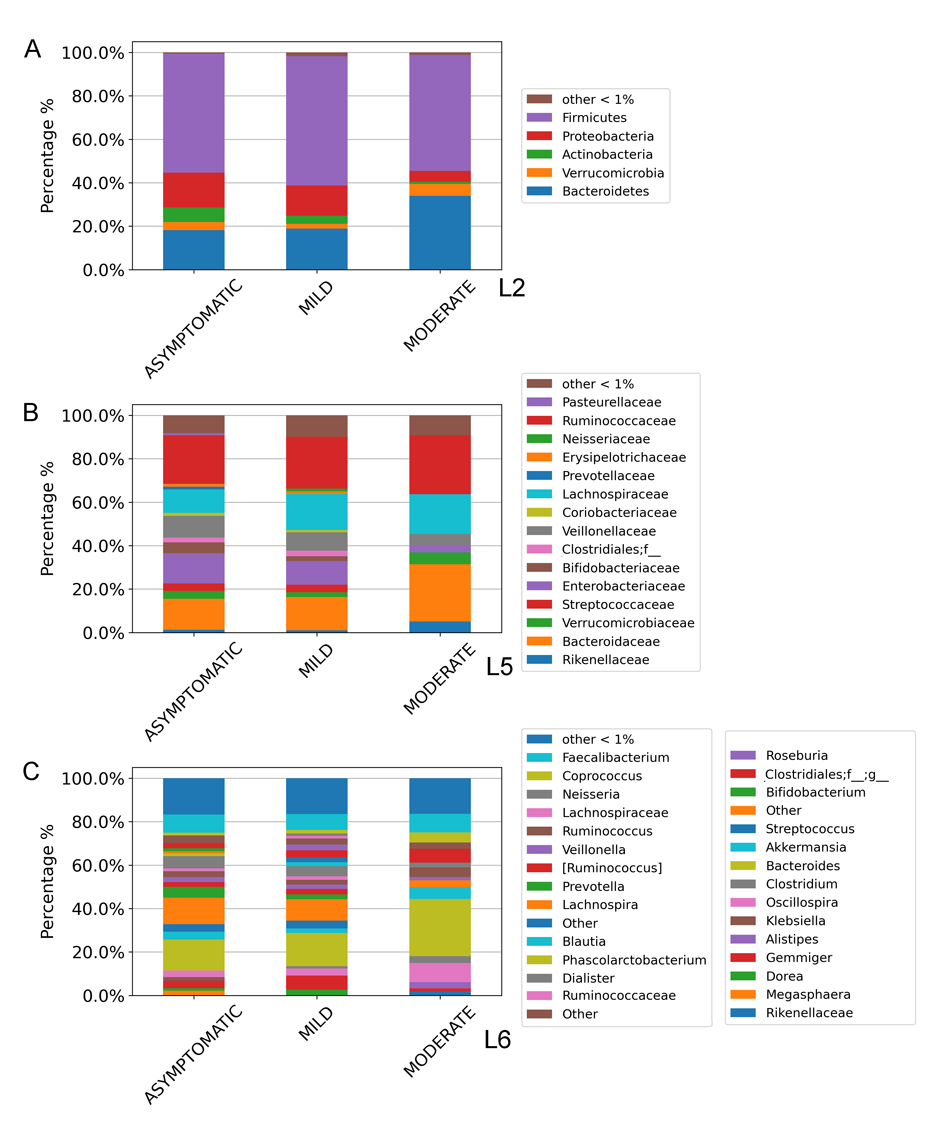


**Figure S9.** ASV distributions of “asymptomatic”, “mild” and “moderate” groups at L2 (A), L5 (B), L6 (C). Only ASV>1% distributions were reported in the comparisons amongst the three groups.


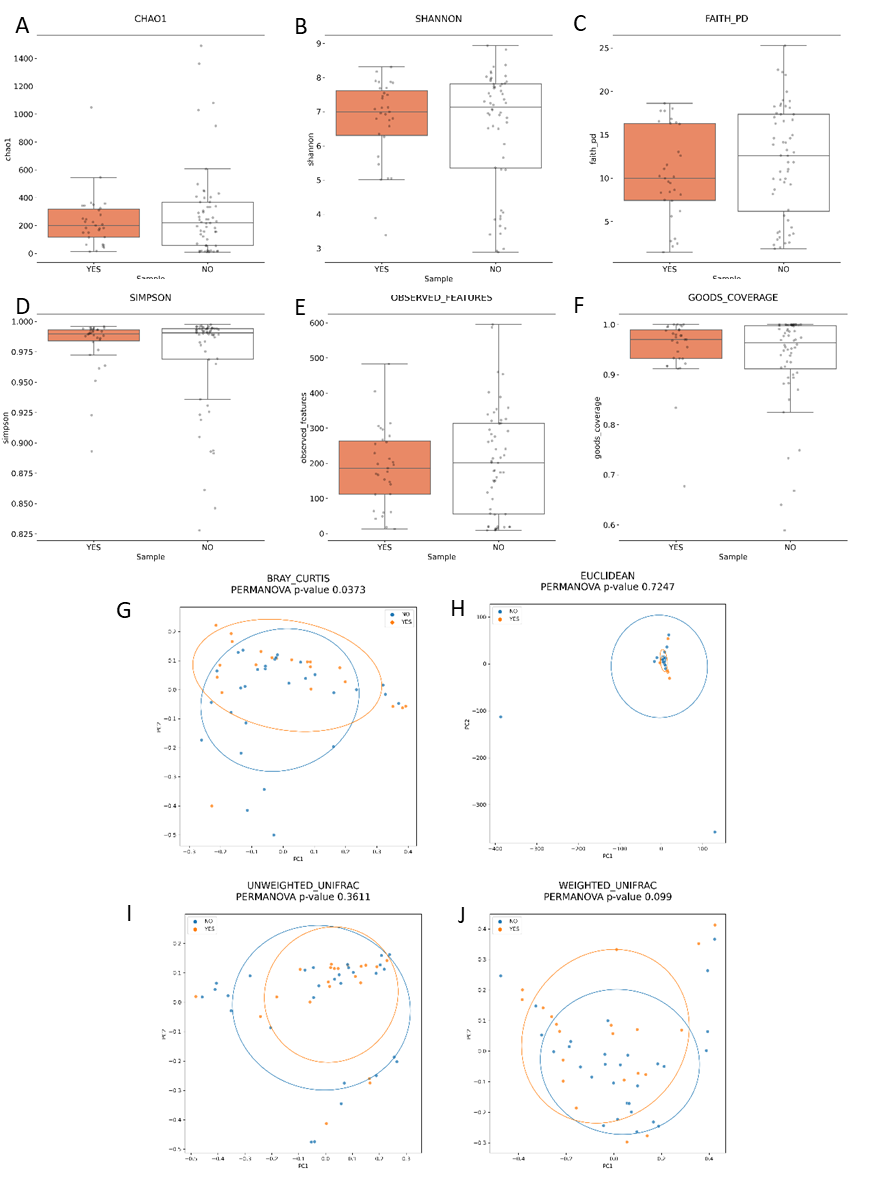


**Figure S10** Evaluation of the α-diversity based on Chao-1 (A), Shannon (B), Faith PD (C), Simpson (D), observed features (E) and Goods coverage (F) indices and β-diversity performed by Bray-Curtis (G), Euclidian distance (H), unweighted (I) and weighted UniFrac (J) algorithms between COVID-19 who received the antibiotic (YES) and COVID-19 patient who did not (NO).


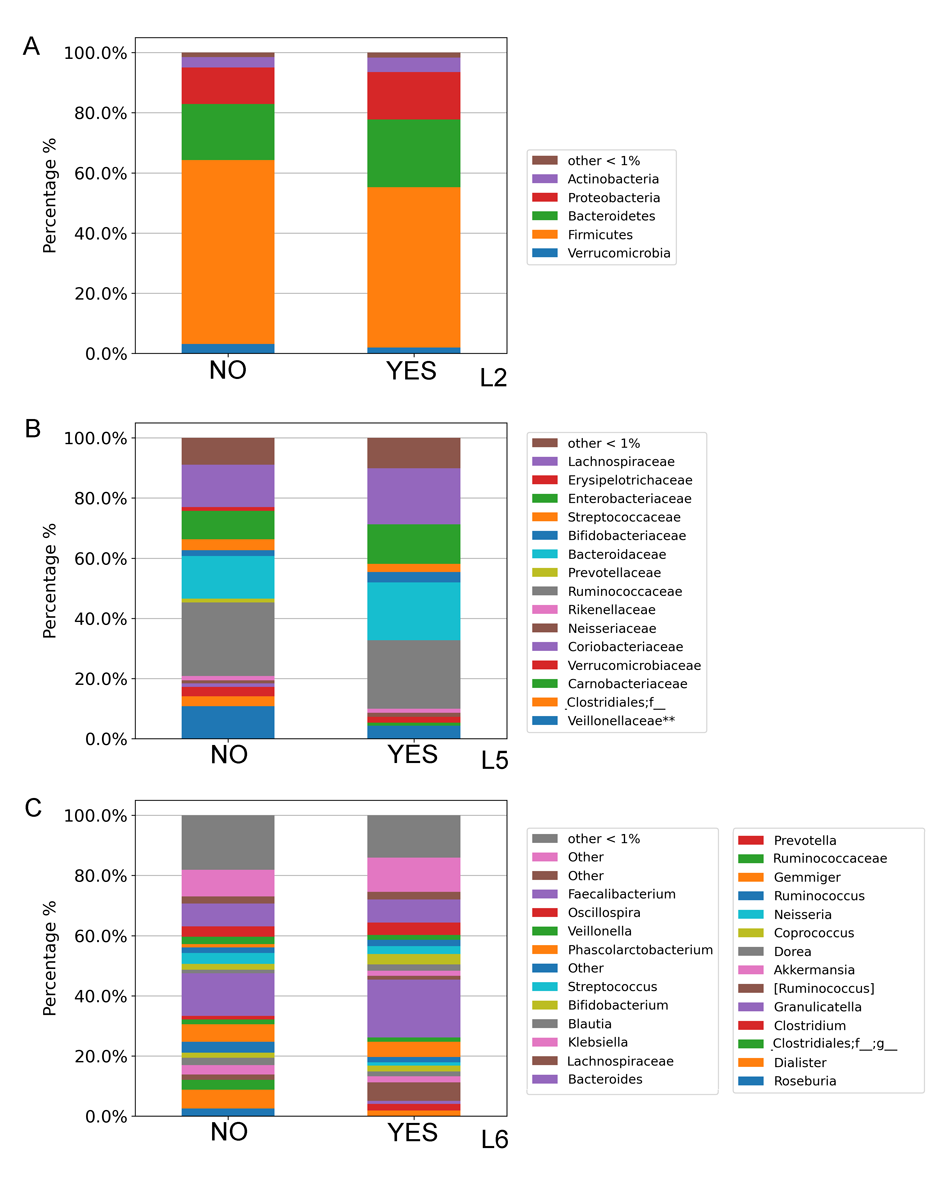


**Figure S11.** ASV distribution at L2 (A), L5 (B), L6 (C) of COVID-19 who received the antibiotic and COVID-19 patient who did not. Only ASV>1% distributions were reported in the comparisons amongst the three groups.


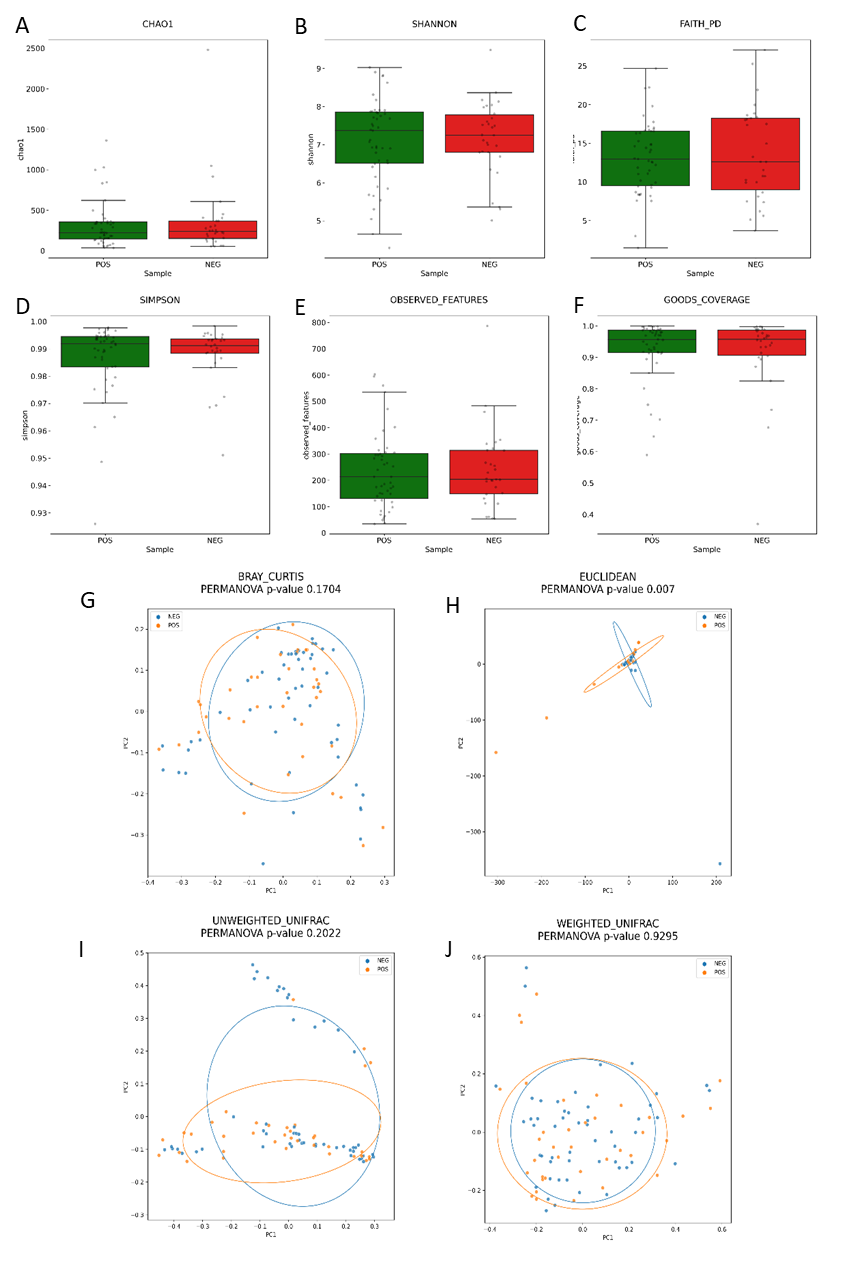


**Figure S12.** Evaluation of the α-diversity based on Chao-1 (A), Shannon (B), Faith PD (C), Simpson (D), observed features (E) and Goods coverage (F) indices and β-diversity performed by Bray-Curtis (G), Euclidian distance (H), unweighted (I) and weighted UniFrac (J) algorithms between “Sars-CoV-2 stool positive group” and “Sars-CoV-2 stool negative group”.


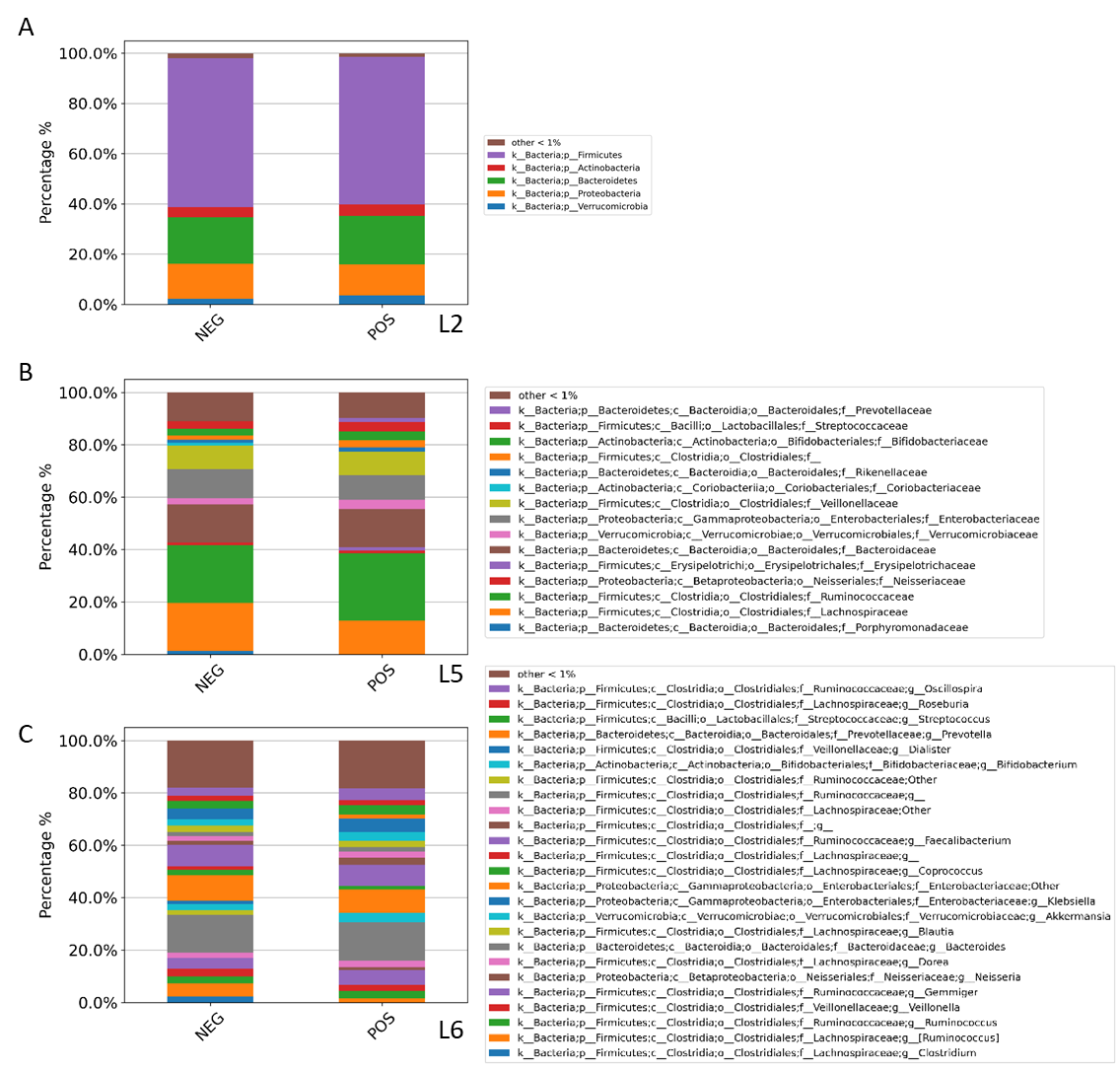


**Figure S13** ASV distribution at L2 (A), L5 (B), L6 (C) of “Sars-CoV-2 stool positive group” and “Sars-CoV-2 stool negative group”. Only ASV>1% distributions were reported in the comparisons amongst the three groups.


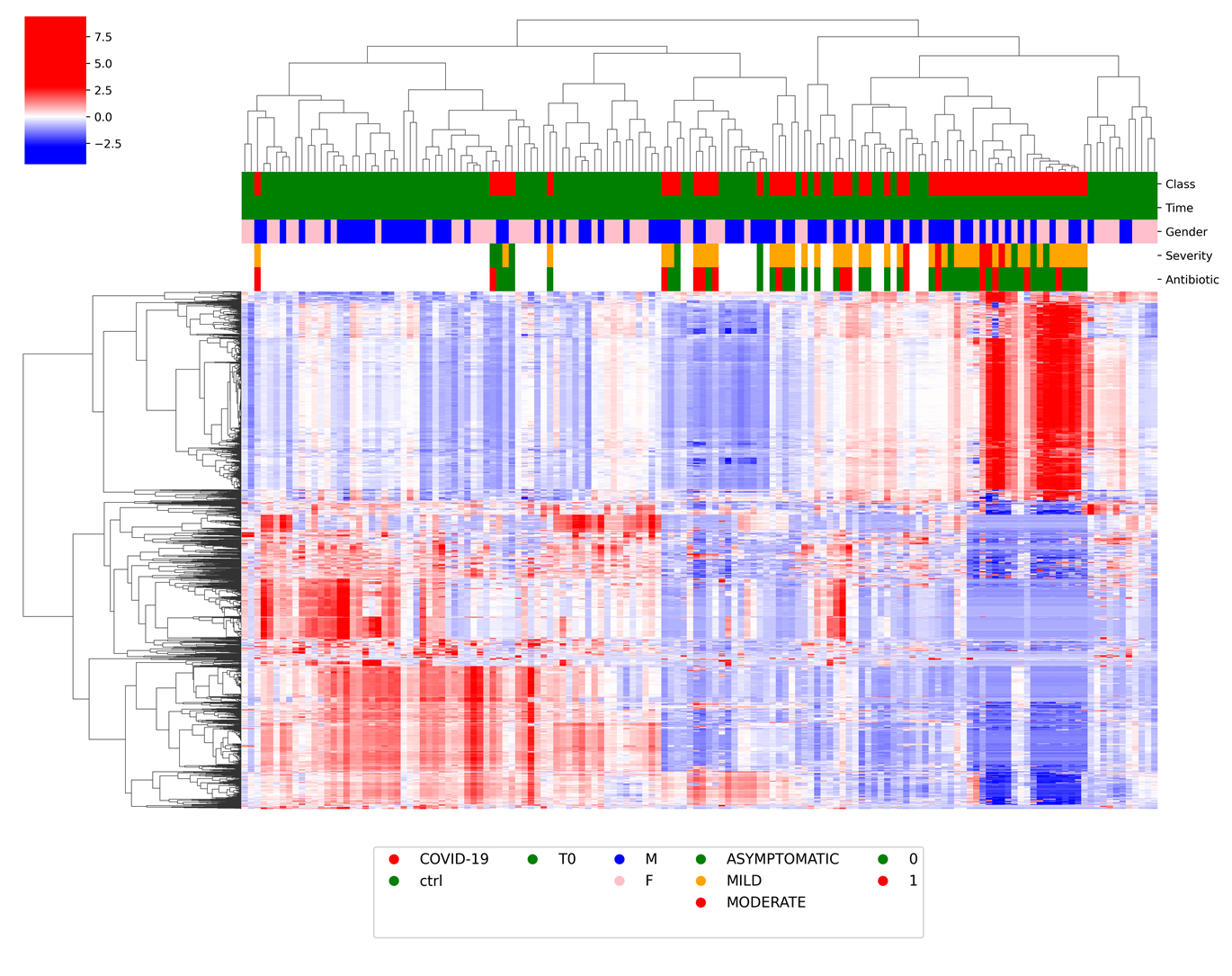


**Figure S14.** Graphical representation of hierarchical analysis of the KO-based prediction of functional profile of microbial communities of COVID-19 and CTRL subgroups highlighted 2148 KOs classification features filtered by a t-test between classes with *p*-value <0.05. The color scale characterizes the Z- score for each variable: red, high level; blue, low level. The column color labels represent respectively: patient’s class (red=COVID-19, orange=Non-COVID-19, green=CTRLs), time (green=T_0_), gender (blue=male, pink=female), severity (green=asymptomatic, orange=mild, red=moderate), and antibiotic (green=absent (0), red=present (1)).

**
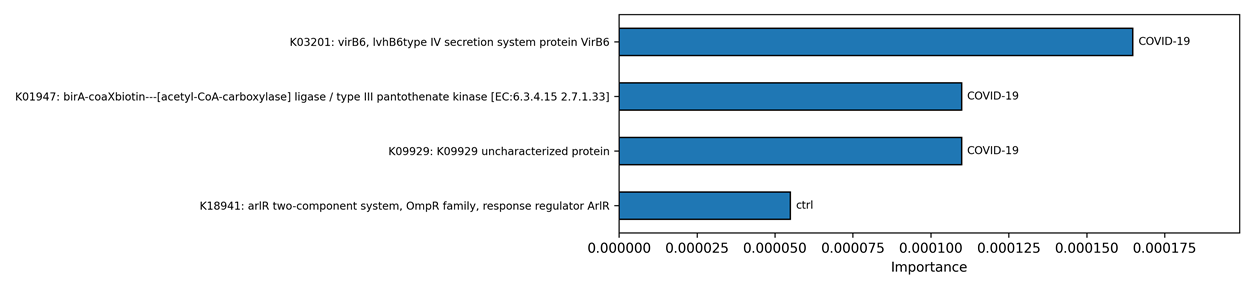
**

**Figure S15. Important KO selected by model classification analysis.** The bars represent the importance scores of each KO’s in the prediction of models.


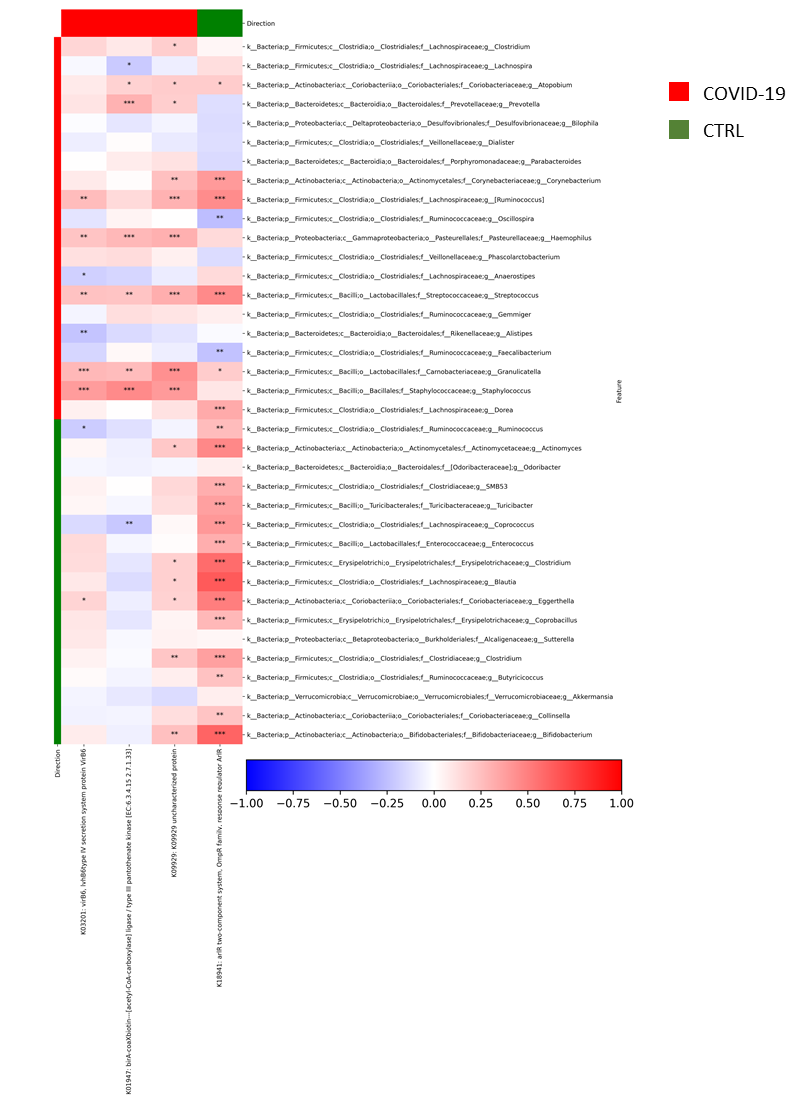


**Figure S16. Pearson’s correlation heatmap between KEGG orthology (KO) and microbiota features at genus level.** The color scale characterizes the correlation values for each variable: red, high level; blue, low level. The column color labels represent respectively “red” COVID-19 patient and “green” controls samples. p<0.001, with FDR<0.05 and FDR<0.001 are represented by *, ** and *** symbols, respectively.

**
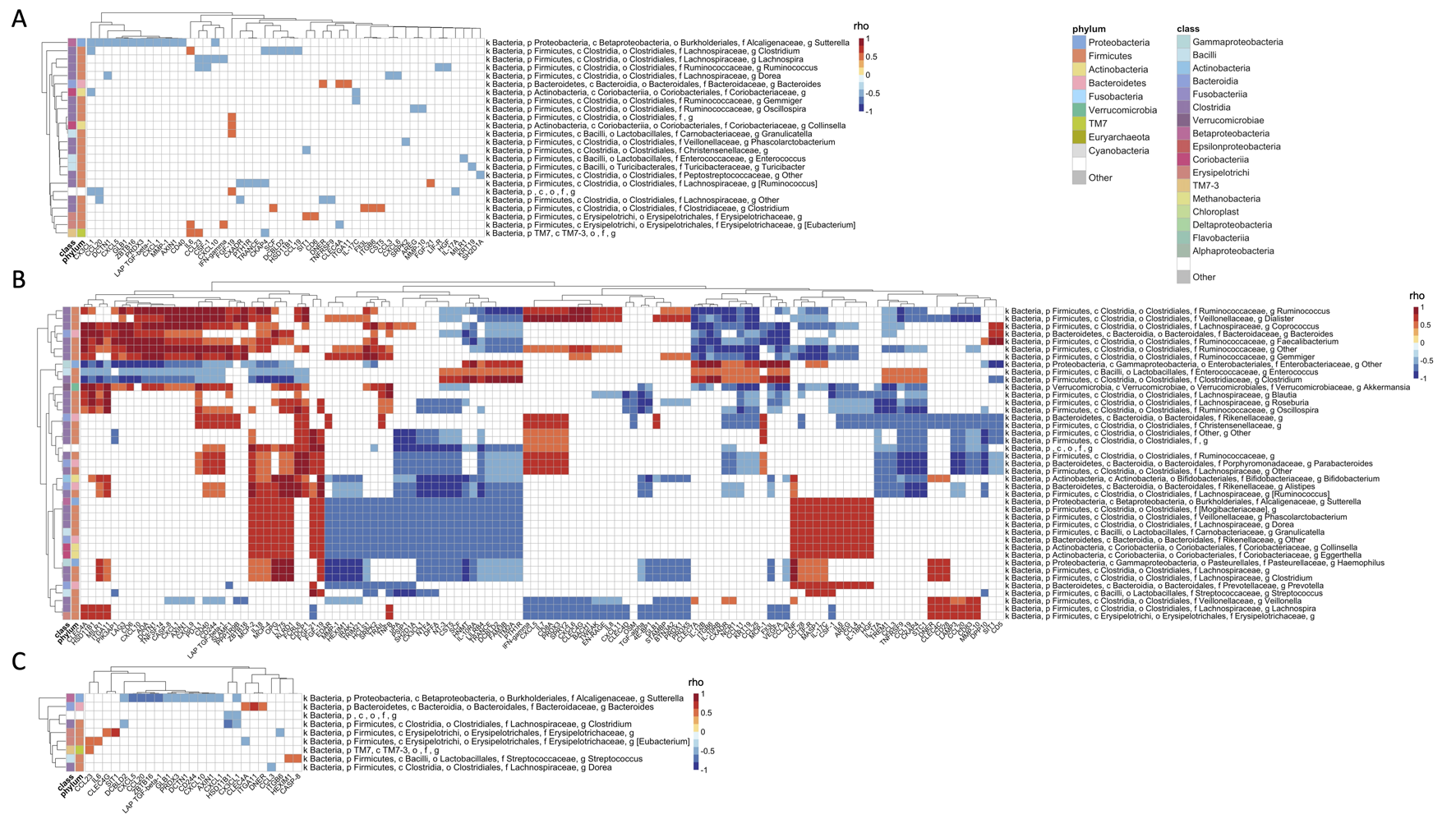
**

**Figure S17**. Heatmap showing Spearman’s correlations between ASVs abundance at L6 and blood proteins for the entire COVID-19 cohort (A), “asymptomatic” (B) and “mild” (C) groups. Red boxes indicate positive correlations, blue boxes show negative correlations and white cells show not statistically significant correlations (FDR >= 0.05).
